# FMO rewires metabolism to promote longevity through tryptophan and one carbon metabolism in *C. elegans*

**DOI:** 10.1038/s41467-023-36181-0

**Published:** 2023-02-02

**Authors:** Hyo Sub Choi, Ajay Bhat, Marshall B. Howington, Megan L. Schaller, Rebecca L. Cox, Shijiao Huang, Safa Beydoun, Hillary A. Miller, Angela M. Tuckowski, Joy Mecano, Elizabeth S. Dean, Lindy Jensen, Daniel A. Beard, Charles R. Evans, Scott F. Leiser

**Affiliations:** 1 Department of Molecular and Integrative Physiology, University of Michigan, Ann Arbor, MI 48109 USA; 2 Cellular and Molecular Biology Program, University of Michigan, Ann Arbor, MI 48109 USA; 3 Department of Internal Medicine, University of Michigan, Ann Arbor, MI 48109 USA

**Keywords:** Ageing, Metabolomics, Metabolomics

## Abstract

Flavin containing monooxygenases (FMOs) are promiscuous enzymes known for metabolizing a wide range of exogenous compounds. In *C. elegans*, *fmo-2* expression increases lifespan and healthspan downstream of multiple longevity-promoting pathways through an unknown mechanism. Here, we report that, beyond its classification as a xenobiotic enzyme, *fmo-2* expression leads to rewiring of endogenous metabolism principally through changes in one carbon metabolism (OCM). These changes are likely relevant, as we find that genetically modifying OCM enzyme expression leads to alterations in longevity that interact with *fmo-2* expression. Using computer modeling, we identify decreased methylation as the major OCM flux modified by FMO-2 that is sufficient to recapitulate its longevity benefits. We further find that tryptophan is decreased in multiple mammalian FMO overexpression models and is a validated substrate for FMO-2. Our resulting model connects a single enzyme to two previously unconnected key metabolic pathways and provides a framework for the metabolic interconnectivity of longevity-promoting pathways such as dietary restriction. FMOs are well-conserved enzymes that are also induced by lifespan-extending interventions in mice, supporting a conserved and important role in promoting health and longevity through metabolic remodeling.

## Abstract (web summary)

Flavin containing monooxygenase 2 (FMO-2) is known to increase lifespan under dietary restriction through incompletely understood mechanisms. Here the authors report that FMO-2 modifies tryptophan and methionine metabolic pathways to enhance stress resistance and slow aging in *C. elegans*.

## Introduction

Flavin-containing monooxygenases (FMOs) are a family of enzymes that oxygenate substrates with nucleophilic centers, such as nitrogen and sulfur^1^. They were first discovered 50 years ago and have been studied extensively under the context of xenobiotic and drug metabolism^1^. FMOs bind to an FAD prosthetic group and interact with an NADPH cofactor to oxygenate substrates^2^. The FMO protein family is highly conserved both genetically and structurally from bacteria to humans^2,3^. Considering the conserved nature of FMOs, it is plausible that they share an endogenous role in addition to detoxifying xenobiotics.

Through a screen of genes downstream the hypoxia-inducible factor-1 (HIF-1), whose stabilization leads to lifespan extension in *C. elegans*, flavin-containing monooxygenase-2 (*fmo-2*) was identified as necessary for the longevity and health benefits of both hypoxia and dietary restriction (DR)^4^. The *fmo-2* gene is also sufficient to confer these benefits on its own when overexpressed^4^. Recently, studies also suggest potential endogenous role(s) for mammalian FMOs, where changes in expression of multiple FMO proteins affect systemic metabolism^5–10^. Initial correlative reports also link FMOs to the aging process, showing that *Fmo* genes are frequently induced in long-lived mouse models, such as DR mice^5,6^. However, the mechanism(s) for how *Fmo*s modulate endogenous metabolism and/or aging in vivo is unclear, as is their potential to benefit health and longevity in multiple species.

While frequently implicated in cancer cells, recent studies identify one carbon metabolism (OCM) as a common downstream target of multiple longevity pathways^11–14^. OCM is an important intermediate metabolic pathway and refers to a two-cycle metabolic network including the folate cycle and the methionine cycle^15^. OCM takes nutrient inputs, including glucose and vitamin B12, and utilizes them to synthesize intermediates for metabolic processes involved in growth and survival, including nucleotide metabolism, the transsulfuration and transmethylation pathways, and lipid metabolism^12,13,16^. In particular, suppressing expression of the methionine cycle gene *sams-1* by RNA-mediated interference (RNAi) extends the wild-type worm lifespan, but fails to further extend the lifespan of the genetic DR model *eat-2* mutants^17^.

Kynurenine synthesis from tryptophan and subsequent metabolism is another important metabolic pathway that can play a role in many processes, including longevity regulation. Knocking out tryptophan 2,3-dioxygenase (TDO), which catalyzes the first and rate-limiting step of this pathway, leads to lifespan extension in worms and flies^18,19^. Similarly, suppressing the kynurenine pathway by knocking down kynureninase (*kynu-1)* in worms also increases lifespan^20^. The kynurenine pathway competes for tryptophan with the serotonergic biosynthesis pathway and produces nicotinamide adenine dinucleotide (NAD) and other metabolites, including kynurenic acid and picolinic acid^21^.

Given that (1) induction of Fmos correlates with increased longevity across species, (2) nematode *fmo-2* is necessary and sufficient to improve health and longevity downstream of metabolic perturbations, such as DR, and (3) loss of Fmo expression can modify aspects of metabolism, we hypothesized that Fmos affect aging by modifying one or more distinct metabolic processes.

In this work, we sought to determine the metabolic changes that occur when the expression of nematode *fmo-2* is perturbed to identify its mechanism of longevity regulation. Our resulting data support a model where *fmo-2* oxygenates tryptophan, leading to alteration of OCM components that confer longevity and healthspan benefits by reducing flux through methylation processes.

## Results

### Fmo-2 alters one carbon metabolism

**Fig. 1:**
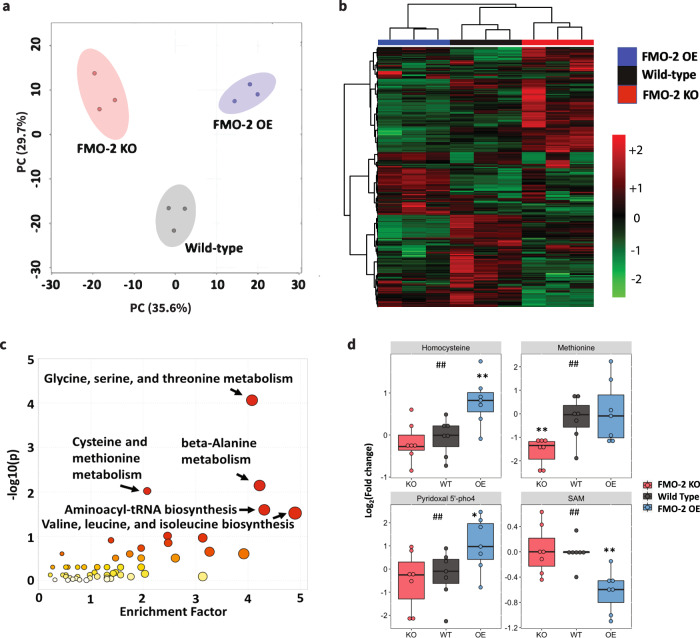
One carbon metabolism is altered by *fmo-2* expression level. **a** Principal component analysis of untargeted LC-MS metabolomics data of wild type, FMO-2 OE, and FMO-2 KO strains of *C. elegans*. **b** Heatmap of untargeted LC-MS metabolomics data of the wild type, FMO-2 OE and FMO-2 KO. The red color indicates metabolites with a higher abundance, while the green color indicates those with a lower abundance. **c** Pathway enrichment analysis using untargeted LC-MS metabolomics data of wild type and FMO-2 OE. The size and the color of the bubbles represent the enrichment factor and the *p*-value respectively for the pathways. The darker the color, the more significant the *p*-value. And larger the radius of the bubble, the greater the enrichment score. **d** Comparison of targeted metabolomics data of metabolites related to OCM between the wild type (black), FMO-2 OE (blue), and FMO-2 KO (red), normalized to the average of wild type intensity. pho4 = phosphate. SAM = s-adenosylmethionine. * represents *p* < 0.05, ** represents *p* < 0.01, and *** represents *p* < 0.001 using two-tailed Student’s unpaired t-test. # represents *p* < 0.05 and ## represents *p* < 0.01 using one-way ANOVA trend analysis. *n* = 7 biologically independent experiments. For t-test (represented by *) *p*-value = 0.00854 (WT vs FMO-2 OE, Homocysteine), 0.0063 (WT vs FMO-2 KO, Methionine), 0.0449 (WT vs FMO-2 OE, Pyridoxal 5′-pho4), 0.00144 (WT vs FMO-2 OE, SAM). For trend analysis (represented by #), *p*-value = 0.0016 (Homocysteine), 0.0059 (Methionine), 0.0213 (Pyridoxal 5′-pho4), 0.0013 (SAM). In box plots, the median is shown by the center line. The upper boundary of the box represents the 75% interquartile range, while the lower boundary represents the 25% interquartile range. **a**–**c** are generated using MetaboAnalyst. Statistics for c and d are in Supplementary Table 1 and 2, respectively No notes = Not significant.

Based on the conserved enzymatic mechanism^2,3^ and our published data supporting a key role for nematode FMO-2 in regulating stress resistance, healthspan and longevity^4^, we hypothesized that FMO-2 may significantly alter endogenous metabolism in *C. elegans*. To test if systemic metabolism was broadly altered by FMO-2, we used untargeted metabolomics analysis (Supplementary Data 1) of three strains with varying *fmo-2* expression: the wild-type reference strain (N2 Bristol), the *fmo-2(ok2147)* putative knockout strain (FMO-2 KO), and our previously published long-lived *fmo-2* overexpression (KAE9) strain (FMO-2 OE). The resulting principal component analysis (PCA) shows a substantial explained variance (65.3%) through principal components (PC) 1 and 2 (Fig. 1a). Our untargeted metabolomics data suggest a distinct difference in the metabolome between the three strains, consistent with expression of nematode *fmo-2* being sufficient to modify endogenous metabolism (Fig. 1b).

Having established broadly that *fmo-2* expression modifies metabolism, we next asked what key metabolic aspects are modified. Using *p*-value <0.05 as our significance threshold, we identified five metabolic pathways that are significantly altered by the overexpression of *fmo-2*, most of which are involved in amino acid metabolism (Fig. 1c, Supplementary Table 1). Of the five pathways, we observed the most significant enrichment in glycine, serine, and threonine metabolism (Fig. 1c). Exogenous supplementation of glycine in worm diet is reported to extend lifespan by remodeling the methionine cycle^22^, a component of one carbon metabolism (OCM) and another significantly enriched metabolic pathway from our analysis, cysteine and methionine metabolism (Fig. 1c, Supplementary Table 1). Indeed, OCM is a nexus of multiple metabolic pathways that are necessary for survival; OCM is implicated in multiple longevity pathways, including dietary restriction, insulin/IGF-1 signaling, and the metformin-induced longevity response^13,16,23^. Due to its relevance in multiple longevity pathways and the direct involvement of cysteine and methionine metabolism within this metabolic network, we postulated that *fmo-2* regulates longevity through its interactions with OCM.

To test whether *fmo-2* expression modifies OCM, we used targeted metabolomics analysis on a panel of metabolites involved in OCM and related pathways to determine whether their abundance levels were altered following *fmo-2* expression (Supplementary Data 3). We hypothesized that the affected metabolites would have abundance levels that correlate with *fmo-2* expression level. To look for changes between groups, we initially compared the metabolite levels between the wild type and FMO-2 OE and also between the wild type and FMO-2 KO. In line with our hypothesis that OCM is altered by *fmo-2* expression, we observed significant changes in abundance levels of homocysteine, s-adenosylmethionine (SAM), cystathionine and pyridoxal 5′-phosphate in FMO-2 OE, and of methionine in FMO-2 KO, when compared to the wild type (Supplementary Table 2). To test whether *fmo-2* expression levels statistically modulate OCM metabolites in correlation with *fmo-2* expression, we performed ANOVA trend analysis on the data from all three strains. We identified a significant change in levels of methionine, pyridoxal 5′-phosphate, homocysteine, and SAM in line with changes in the expression of *fmo-2* (Fig. 1d). Furthermore, betaine, folic acid, serine, and cystathionine levels all show a trend with *fmo-2* expression, but they did not reach statistical significance below a 0.05 *p*-value (Supplementary Fig. 1). Taken together, our results are consistent with the hypothesis that the OCM pathway is modified by *fmo-2* expression.

### One carbon metabolism interacts with *fmo-2* to regulate stress resistance and longevity

**Fig. 2:**
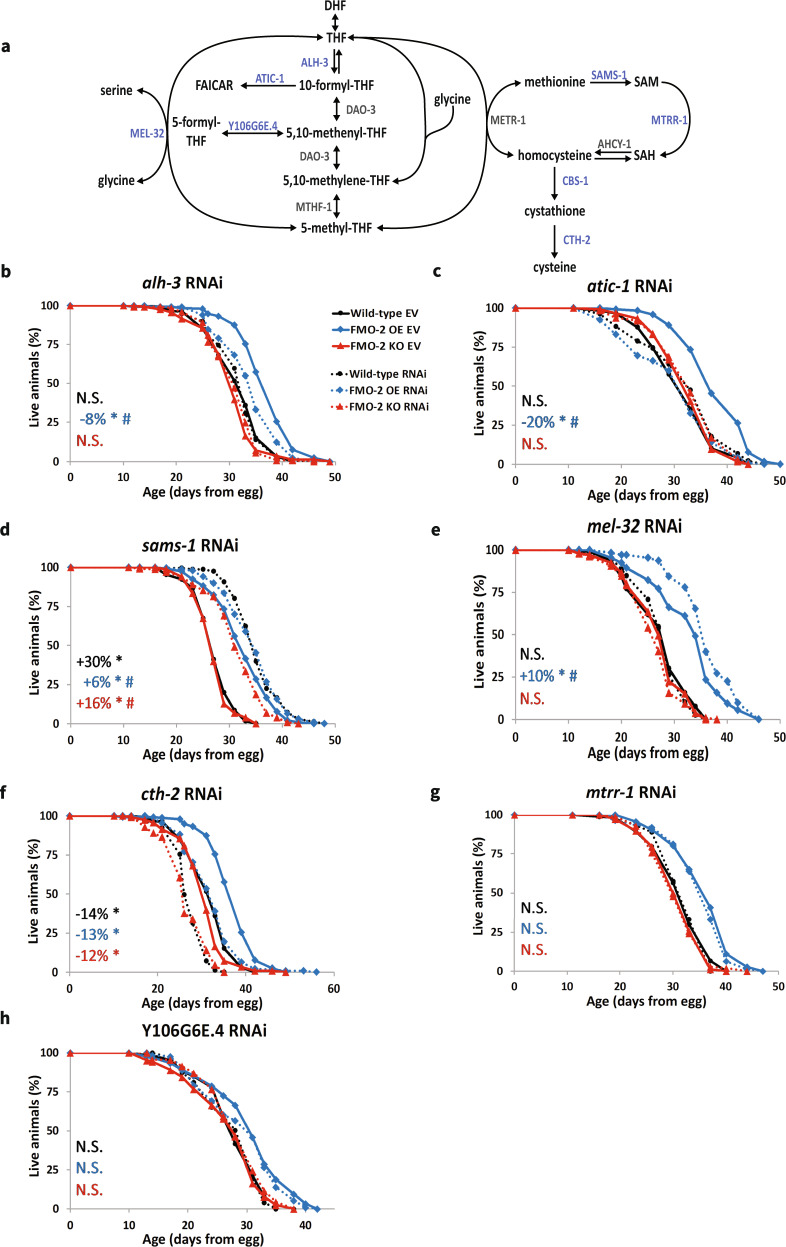
*Fmo-2* interacts with OCM genes to regulate lifespan. **a** Diagram of OCM network. Genes included here are labeled in blue and genes not included are labeled in gray. Lifespan analysis comparing the wild type, FMO-2 OE and FMO-2 KO on empty-vector (EV) and **b** *alh-3* RNAi, **c** *atic-1* RNAi, **d** *sams-1* RNAi, **e** *mel-32* RNAi, **f** *cth-2* RNAi, **g** *mtrr-1* RNAi, and **h** Y106G6E.4 RNAi. Percent change in mean lifespan compared to their respective EV controls are shown in the left bottom corner of the figures. Black circle = wild type, blue diamond = FMO-2 OE, and red triangle= FMO-2 KO. Solid line = EV and dotted line = RNAi. * denotes significant change in mean lifespan at *p* < 0.05 using log-rank and # denotes significant interaction between the RNAi of interest and *fmo-2* genotype at *p* < 0.01 using Cox regression analysis. N.S. = not significant. Statistics are in Table 1, Supplementary Table 5, and Supplementary Data 5.

Having established that FMO-2 modifies endogenous metabolism broadly and OCM specifically, we next hypothesized that these metabolic changes are causal for longevity phenotypes. Previous studies identify increased stress resistance as a common phenotype shared by multiple long-lived organisms both within and between species^24–27^ and *fmo-2* is known to be highly regulated at the transcript level by many stresses^28^. To determine the functional interaction between *fmo-2* and OCM, we used RNAi to knockdown the expression of genes involved in OCM (Fig. 2a) and tested for their role in promoting or repressing survival against the oxidative stressor paraquat. Of the eight genes that we tested, the individual knockdown of four genes, *sams-1*, *mel-32*, *mtrr-1*, and *Y106G6E.4*, exhibit altered stress resistance phenotypes for the wild type and FMO-2 OE (Supplementary Fig. 2A–D), as assessed using log-rank test with a cutoff threshold of *p* < 0.05 compared to the empty vector (EV) controls. Two of these genes, *sams-1*, and *mel-32*, show interaction with FMO-2 OE, as assessed by Cox regression analysis with *p*-value cutoff of 0.01 (Supplementary Table 3). We note that 7 of 8 genes showed significant (*p* < 0.05) knockdown via RT-QPCR and the 8th *p* = 0.066, with most genes knocked down by at least 70% by their individual RNAi (Supplementary Fig. 3). Interestingly, while *sams-1* knockdown extends worm lifespan^17^, we find that knocking down *sams-1* decreases the stress resistance of the wild type and FMO-2 OE (Supplementary Fig. 2A), suggesting that the regulation of lifespan and stress resistance are uncoupled in this instance, as have been reported previously^29^. This result is similar to previous work showing that *sams-1* knockdown is detrimental to survival under pathogen exposure^30^. We further find that *sams-1* knockdown interacts with FMO-2 OE, whereby it more severely decreases FMO-2 OE paraquat resistance compared to that of the wild type (Supplementary Fig. 2A). Conversely, we find that knocking down *mel-32* increases the resistance of both the wild type and FMO-2 OE, but it again shows an interaction with FMO-2 OE, whereby FMO-2 OE has more modest extension compared to the wild type (Supplementary Fig. 2B).

We also find that knocking down *Y106G6E.4* and *mtrr-1* increase the stress resistance of both the wild type and FMO-2 OE without significant interaction with FMO-2 OE (Supplementary Fig. 2C, D), suggesting that the stress resistance conferred by the suppression of these genes is independent of *fmo-2*. Knocking down *atic-1*, *alh-3, cbs-1,* and *cth-2* yield inconsistent results in our experiments (Supplementary Table 4). Overall, our data show that 2/8 of the genes that we tested interact with FMO-2 OE to modify paraquat resistance. While these results do not definitively prove that FMO-2 acts through OCM, they are consistent with the hypothesis that OCM is a regulator of stress resistance and that there is a genetic interaction between *fmo-2* and OCM in that regulation.

To test the interaction between *fmo-2* and OCM more directly, we performed lifespan experiments using RNAi knockdown of genes from our paraquat resistance screen (Fig. 2b–h). We included FMO-2 KO in the lifespan analysis to determine if the interactions that we identify are dependent on *fmo-2* expression. Multiple gene knockdowns show altered lifespan phenotypes for the wild type, FMO-2 OE, and FMO-2 KO that suggest interaction with FMO-2. Of the eight genes we tested, knockdown of two genes, *alh-3* and *atic-1*, suppress the lifespan extension of FMO-2 OE without affecting the lifespan of the wild type and FMO-2 KO (Fig. 2b, c). Similar to our paraquat stress resistance experiments, we conclude this based on (1) log-rank test showing changes only in FMO-2 OE lifespan with a cutoff threshold of *p* < 0.05 compared to their respective empty vector (EV) controls, and (2) cox regression analysis (Supplementary Table 5, Table 1) showing a differential interaction with FMO-2 OE and FMO-2 KO, using a cutoff threshold of *p* < 0.01. These results suggest that *alh-3* and *atic-1* are required for *fmo-2*-mediated lifespan extension and are consistent with previous reports that their expression levels are upregulated in long-lived worms^11,31^. *alh-3* is upregulated in *eat-2* mutants and *atic-1* is upregulated in both *eat-2* and *daf-2* mutants^11,31–33^. In addition, *atic-1* is involved in the conversion of 10-formyl-THF to formyl-5-aminoimidazole-4-carboxamide-1-beta-4-ribofuranoside (FAICAR) (Fig. 2a). FAICAR inhibits the synthesis of AICAR^34^, which is a molecule reported to induce the phosphorylation and activation of AMP-activated protein kinase (AMPK)^35^, whose activation extends lifespan^36^. Therefore, suppressing *atic-1* expression may reduce FMO-2 OE lifespan by inhibiting AMPK activation. This potentiates the possibility that AMPK may also be involved in the FMO-2-mediated longevity regulation. In sum, it is plausible that these genes are required for multiple longevity pathways, including FMO-2 overexpression.

**Table 1:** Cox regression analysis of OCM/tryptophan metabolism genes and *fmo-2* modified strains lifespan data

	Experimental		FMO-2 KO		FMO-2 OE		Interact with FMO-2 KO		Interact with FMO-2 OE	
	Haz. ratio	*p*-value	Haz. ratio	*p*-value	Haz. ratio	*p*-value	Haz. ratio	*p*-value	Haz. ratio	*p*-value
*alh-3*	0.974	0.715	1.050	0.510	0.480	<0.001	1.088	0.413	1.333	0.005
*atic-1*	0.923	0.269	1.051	0.501	0.511	<0.001	0.898	0.302	1.641	<0.001
*sams-1*	0.298	<0.001	1.003	0.972	0.359	<0.001	1.632	<0.001	2.652	<0.001
*mel-32*	1.098	0.246	1.110	0.191	0.505	<0.001	0.891	0.307	0.455	<0.001
*cth-2*	1.619	<0.001	1.122	0.072	0.491	<0.001	1.183	0.058	0.932	0.417
*mtrr-1*	1.184	0.025	1.152	0.058	0.490	<0.001	0.963	0.718	1.089	0.421
*Y106G6E.4*	0.949	0.470	1.053	0.485	0.481	<0.001	0.848	0.118	1.079	0.456
*kmo-1*	3.160	<0.001	1.389	<0.001	0.513	<0.001	0.678	<0.001	0.798	0.038
*tdo-2*	0.306	<0.001	1.388	<0.001	0.510	<0.001	1.464	0.001	1.830	<0.001
*nkat-1*	1.090	0.221	1.298	<0.001	0.512	<0.001	0.836	0.067	0.785	0.015
Formate	0.623	<0.001	1.320	0.005	0.403	<0.001	1.231	0.156	1.625	0.001

Experimental = effect of experimental condition on worm lifespan; FMO-2 KO = effect of knocking out *fmo-2* on worm lifespan; FMO-2 OE = effect of overexpressing *fmo-2* on worm lifespan; interact with FMO-2 KO = interaction between experimental condition and knocking out *fmo-2* on worm lifespan; interact with FMO-2 OE = interaction between experimental condition and overexpressing *fmo-2* on worm lifespan; Hazard ratio > 1 = decrease in lifespan; Hazard ratio < 1 = increase in lifespan.

In contrast to *alh-3* and *atic-1*, knockdown of *sams-1* increases the lifespan of the wild type, FMO-2 KO, and FMO-2 OE animals (Fig. 2d). However, *sams-1* knockdown shows interactions with both FMO-2 KO and FMO-2 OE, whereby both strains show less extension under *sams-1* knockdown compared to the wild type, with FMO-2 OE showing a larger interaction effect size (Table 1). This suggests that there is a functional overlap between *sams-1* and *fmo-2* in regulating longevity. Additionally, we find that knocking down *mel-32* only interacts with FMO-2 OE and extends its lifespan (Table 1 and Fig. 2e). It is plausible that the metabolic alterations resulting from increased *fmo-2* expression are required for *mel-32* gene suppression to promote worm lifespan.

Knockdown of the remaining four genes, *cbs-1*, *cth-2*, *mtrr-1*, and Y106G6E.4, do not show interaction with FMO-2 KO and FMO-2 OE (Table 1). However, knocking down *cth-2* decreases the lifespan of all three strains, suggesting that this gene is generally required for worm health and survival (Fig. 2f). Knocking down *mtrr-1* and Y106G6E.4 do not affect the lifespan of the worms in our experiments (Fig. 2g, h). Knocking down *cbs-1* yields inconsistent results in our experiments (Supplementary Table 5).

In total, our data show that half (4/8) of the genes tested interact with FMO-2 OE: two genes are required for FMO-2 OE lifespan extension (*alh-3* and *atic-1*), another gene interacts with FMO-2 OE and FMO-2 KO (*sams-1*), placing it in the same functional pathway with FMO-2, and one gene only extends the lifespan of FMO-2 OE when knocked down (*mel-32*). Together, our lifespan data, combined with our metabolomics results, support an interaction between *fmo-2* and genes involved with OCM in regulating worm lifespan.

### Fmo-2 influences longevity by modulating the transmethylation pathway

Our data are consistent with a model where *fmo-2* interacts with OCM to regulate longevity and stress resistance. Previous studies identify multiple pathways that affect longevity and are also involved in OCM, including nucleotide metabolism, the transsulfuration pathway, and the transmethylation pathway^11,16,17^. Some of these pathways are also implicated in modifying longevity downstream of dietary restriction in multiple animal models^16,17,37^, making it likely that one or more of these pathways are in the same functional pathway as *fmo-2*. However, the metabolic consequences of *fmo-2* expression on these pathways are not clear based on the changes observed in our targeted metabolomics analysis alone, as the data only show metabolic changes at a single time point and most of the metabolites within OCM are intermediate metabolites. The stress resistance and lifespan results further complicate interpretation as some genes do not affect these phenotypes and some have effects that are independent of *fmo-2*.

**Fig. 3:**
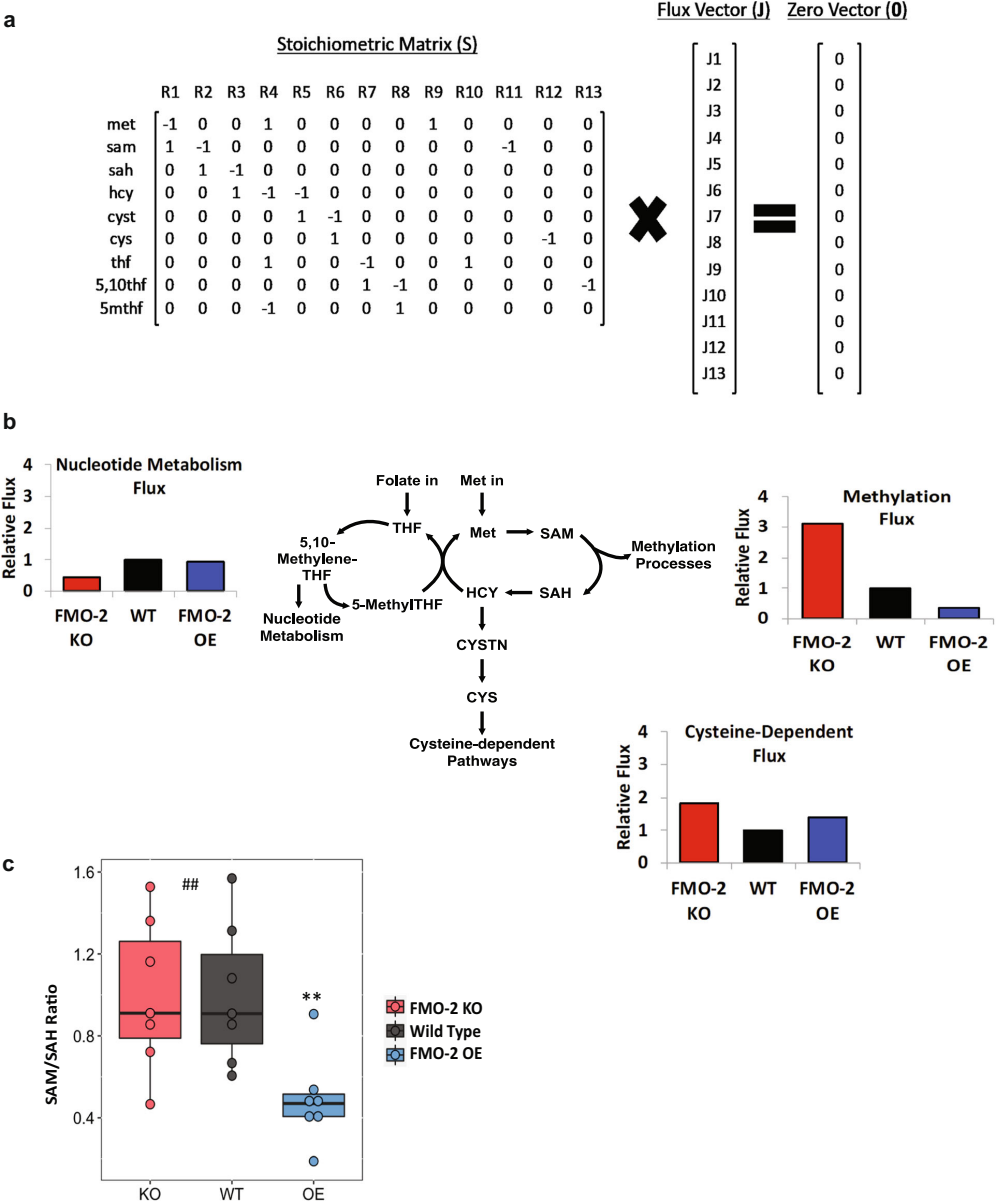
Methylation flux is altered following changes in *fmo-2* expression. **a** Schematic of computational model. **b** Model predictions of output metabolic fluxes. **c** SAM/SAH ratio of the wild type (WT), FMO-2 OE (OE), and FMO-2 KO (KO). Black color = WT, blue color = OE, red color = KO. ** represents *p* < 0.01 using two-tailed unpaired Student’s t-test and ## represents *p* < 0.01 using one-way ANOVA trend analysis. For t-test, *p*-value = 0.0061 (WT vs FMO-2 OE), and for trend analysis, *p*-value = 0.0074. In box plot, the median is shown by the center line. *n* = 7 biologically independent experiments. The upper boundary of the box represents the 75% interquartile range, while the lower boundary represents the 25% interquartile range.

To help determine the biological relevance of the changes we observed in the OCM network following *fmo-2* expression, we applied a computational model (Supplementary Data 6) to predict how enzyme expression (Supplementary Table 6) changes may affect the output fluxes of OCM. The model assumes a steady-state mass balance of fluxes in the reactions illustrated in Fig. 3a. This simple model includes eight reaction fluxes and five fluxes representing transport of methionine (met), tetrahydrofolate (thf), s-adenosylmethionine (sam), cysteine (cys), and 5,10-methylenetetrahydrofolate (5,10thf) into and out of the folate cycle and the methionine cycle. The model output fluxes represent important inputs for the nucleotide metabolism, the transsulfuration pathway, and the transmethylation pathway, each of which are reported to be important for influencing the aging process^11,16,17^ and are potential targets for the *fmo-2*-mediated longevity response. The stoichiometric coefficients for the reaction and transport processes in this system are stored in the matrix S (Supplementary Table 7), where under steady-state conditions S*J = **0**, where **J** is the vector of fluxes^38,39^. The entries in the vector **J** and matrix S are defined in Fig. 3a. Vectors that satisfy the mass-balance relationship S*J = **0** are said to belong to the nullspace of S. To predict how changes in the expression of genes for the enzymes catalyzing the reactions in this network may affect the output fluxes, we projected the gene expression data (Supplementary Table 6) onto the nullspace of S (details provided in the “Methods”). This projection predicts an inverse correlation between *fmo-2* expression and flux through methylation reactions, where the methylation flux is predicted to be reduced in FMO-2 OE and increased in FMO-2 KO compared to wild type (Fig. 3b, Supplementary Fig. 3A–C). This analysis does not predict correlative changes to flux through nucleotide metabolism or the transsulfuration pathway.

Based on this analysis, we hypothesized that artificially decreasing the flux through methylation should replicate FMO-2 OE longevity in the wild type and FMO-2 KO strains, while not affecting the FMO-2 OE worms. *sams-1* encodes for s-adenosylmethionine synthase and is involved in the conversion of methionine into s-adenosylmethionine (SAM). Suppression of *sams-1* has been previously shown to decrease SAM level^40^ and increase longevity^17^. Moreover, while *sams-1* has multiple orthologs, previous work has shown that knocking down *sams-1* is sufficient to manipulate SAM levels in worms^40^. We find that *sams-1* RNAi recapitulates FMO-2 OE lifespan extension in the wild type while interacting with FMO-2 OE, whose lifespan was significantly less affected (Fig. 2d, Table 1). Our data are consistent with previous studies showing that knockdown of *sams-1* fails to further extend the lifespan of the genetic DR model *eat-2* mutants^17^, thereby placing *sams-1* knockdown in the same functional pathway as FMO-2 OE.

To validate the model metabolically, we used the abundance level of SAM and s-adenosylhomocysteine (SAH) from our targeted metabolomics analysis to calculate the SAM/SAH ratio. The SAM/SAH ratio is used as a biomarker for methylation potential, where a decrease in the ratio suggests a hypomethylated state and an increase suggests a hypermethylated state^41,42^. Consistent with our computational model prediction, we observed a significant reduction in the SAM/SAH ratio for FMO-2 OE (hypomethylation) and a significant trend in SAM/SAH ratio corresponding to *fmo-*2 expression by ANOVA trend analysis. (Fig. 3c). In addition, we find that supplementing the worm diet with 2 mM SAM results in a reduction of FMO-2 OE lifespan (Supplementary Fig. 3D). Overall, our computational model prediction and experimental data support the hypothesis that *fmo-2* expression reduces flux through the transmethylation pathway, and that this reduction extends worm lifespan.

### Mammalian FMO metabolomics analysis reveals tryptophan as a substrate of FMO-2

Our data thus far suggest a model where *fmo-2* interacts with OCM to modulate the aging process. However, since FMOs are promiscuous enzymes that oxygenate many nucleophilic atoms, the mechanism by which *fmo-2* induction leads to changes in OCM is not readily evident. FMOs are known as xenobiotic metabolizing enzymes, with many known exogenous targets and few known endogenous targets^1^. Despite extensive knowledge on their enzymatic activity and recent data linking FMOs to endogenous metabolism, no link between specific and systemic metabolism has been made. We hypothesize that a limited number of FMO targets are causal in FMO-2’s effects on OCM and, importantly, on the aging process.

**Fig. 4:**
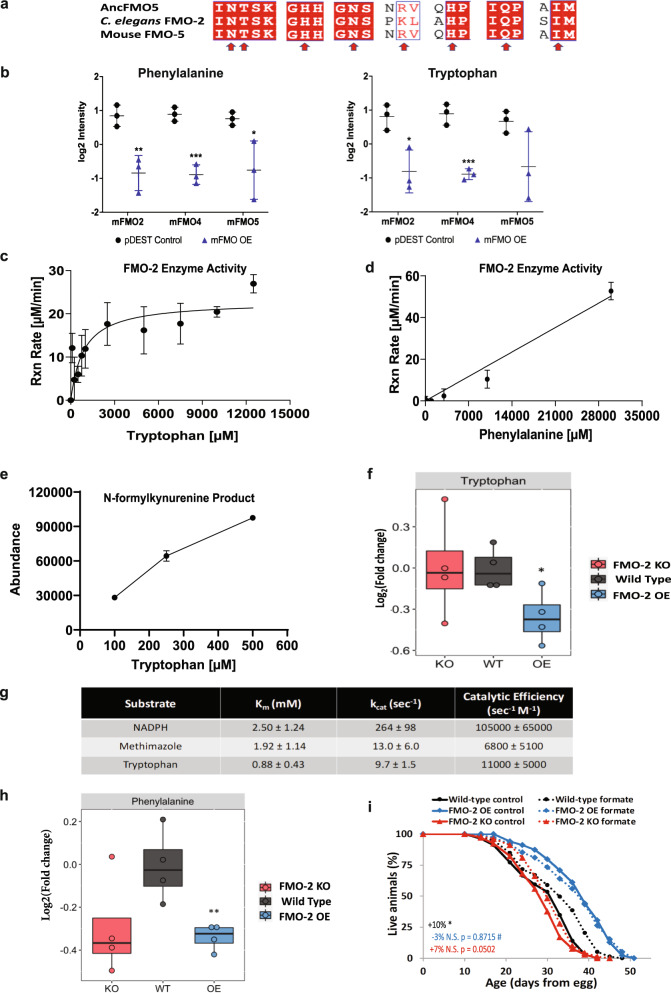
Mammalian FMO metabolomics analysis reveals the tryptophan/kynurenine pathway as a target of FMO-2. **a** Conserved catalytic residues between CeFMO-2 and mFMO5 (indicated by red arrows). **b** The level of phenylalanine and tryptophan present in HepG2 cells expressing pDEST control vector, mFMO2, mFMO4, and mFMO5. Black dot = vector control and blue triangle = FMO OE. * represents *p* < 0.05 by two-tailed unpaired Student’s t-test. For phenylalanine, *p*-value = 0.0084 (control vs mFMO2 OE), 0.00095 (control vs mFMO4 OE), 0.04 (control vs mFMO5 OE). For tryptophan, *p*-value = 0.0188 (control vs mFMO2 OE), 0.000918 (control vs mFMO4 OE), 0.098 (control vs mFMO5 OE). Data is represented as mean values +/− SD, *n* = 3 biological replicates. **c**, **d** The reaction rate by concentration for purified CeFMO-2 enzyme toward tryptophan (*n* = 7 biologically independent replicates) and phenylalanine (*n* = 2 biologically independent replicates) at 30 °C. Data is represented as mean values +/− SD. **e** The abundance of N-formylkynurenine based on LC-MS analysis of CeFMO-2 activity toward 100, 250, and 500 μM tryptophan at 30 °C. *n* = 3 (100 µM and 250 µM Tryptophan) and *n* = 2 (500 µM Tryptophan) biologically independent replicates. Data is represented as mean values +/− SD. **f** Comparison of targeted metabolomics data of tryptophan between the wild type (black), FMO-2 OE (blue), and FMO-2 KO (red), normalized to the average of wild type intensity. *n* = *4* biologically independent replicates and *p*-value = 0.028 (WT vs FMO-2 OE). * = *p* < 0.05 using two-tailed unpaired Student’s t-test. **g** Summary table of Michaelis-Menten parameters for CeFMO-2 cofactor and substrate. **h** Comparison of targeted metabolomics data of phenylalanine between the wild type (black), FMO-2 OE (blue), and FMO-2 KO (red), normalized to the average of wild type intensity. *n* = 4 biologically independent replicates and *p*-value = 0.0097 (WT vs FMO-2 OE). ** = *p* < 0.01 using two-tailed unpaired Student’s t-test. **i** Lifespan assay comparing the survival of the wild type, FMO-2 OE, and FMO-2 KO on control and 1 mM formate supplementation conditions. Black circle = wild type, blue diamond = FMO-2 OE, and red triangle = FMO-2 KO. Solid line = EV, dotted line = formate. * denotes significant change in mean lifespan at *p* < 0.05 using log-rank and # denotes significant interaction between the condition of interest and *fmo-2* genotype at *p* < 0.01 using Cox regression analysis. N.S. = not significant. Statistics are in Supplementary Table 10 and Supplementary Data 5. For box plots in **f** and **h**, the median is shown by the center line. The upper boundary of the box represents the 75% interquartile range, while the lower boundary represents the 25% interquartile range.

Due to the high degree of conservation of catalytic residues between mouse FMOs and CeFMO-2 (Fig. 4a), we referred to our previously published targeted metabolomics of mouse FMO overexpressing (OE) HepG2 cells to determine potential metabolic targets of FMO-2^10^. Using this previously published dataset, our selection criteria for putative substrates of FMO-2 included identifying metabolites that had decreased abundance in at least three of the five FMO OE cell lines to pDEST controls. We used this stringent criteria to identify the most well-conserved targets of FMOs, given that no data exist for CeFMO-2 targets. Using this approach, we identified tryptophan and phenylalanine as potential substrates of FMOs (Fig. 4b). To determine if either of these are substrates of FMO-2, we measured the enzymatic activity of isolated FMO-2 protein in the presence of varying concentrations of tryptophan and phenylalanine. We find that FMO-2 is active toward tryptophan at a reasonable K_m_ and k_cat_ (K_m_: 880 ± 430 µM; k_cat_: 9.7 ± 1.5 s^−1^), suggesting that tryptophan is a viable substrate of FMO-2 (Fig. 4c, Supplementary Table 9). In comparison, rat and invertebrate TDO enzymes with tryptophan demonstrate Km values of 221 µM and 276.5 µM, respectively, while mammalian IDO1 and IDO2 have Km values of 19.1–74 µM and 45.9 mM, respectively^43^, putting the Km value of CeFMO-2 within the range of TDO and IDO proteins. FMO-2 was also active toward phenylalanine, but enzymatic activity did not become apparent until 10 mM, suggesting that phenylalanine is not likely a good endogenous substrate of FMO-2 (Fig. 4d). Since FMO-2 has no previously reported activity toward tryptophan, we used LC-MS with 100, 250, and 500 µM tryptophan under the same enzymatic conditions to determine the product of tryptophan oxygenation. Our resulting data show increasing formation of N-formylkynurenine in a concentration-dependent manner with increasing tryptophan in each of the samples. This result suggests that N-formylkynurenine is a product formed by FMO-2 activity toward tryptophan (Fig. 4e). Consistent with these findings, we observe that tryptophan level is significantly reduced in FMO-2 OE compared to the wild type (Fig. 4f). In addition, we find that tryptophan induces *fmo-2* gene expression in vivo, consistent with our hypothesis that tryptophan is a substrate of FMO-2 (Supplementary Fig. 4). The kinetic parameters of FMO-2 toward NADPH, methimazole, and tryptophan are summarized in Fig. 4g. We also tested additional known substrates of mammalian FMOs, all of which were either poor substrates (e.g., cysteine, phenylalanine, and TMA) or non-substrates of FMO-2 (e.g., 2-heptanone). They are summarized with either the concentration of substrate at which FMO-2 activity is first detected or labeled not determined (N.D.) in Supplementary Table 9. While we have observed that phenylalanine level is also significantly reduced in FMO-2 OE compared to the wild type (Fig. 4h), we believe the effect of FMO-2 on phenylalanine to be indirect as it is a poor substrate in vitro (Fig. 4d).

**Fig. 5:**
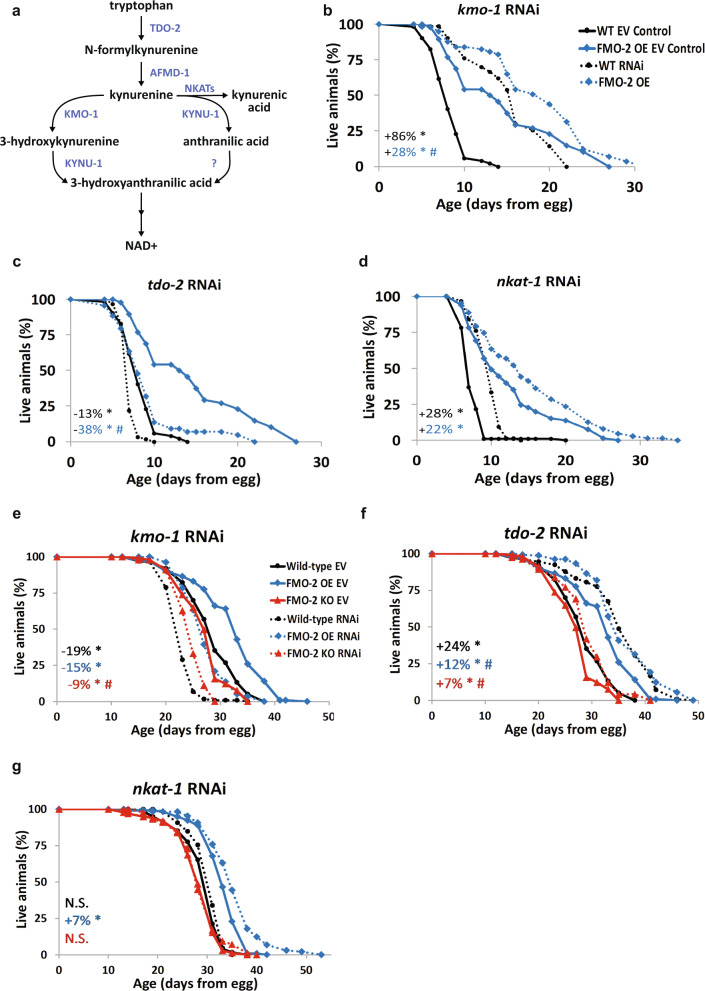
*Fmo-2* interacts with kynurenine metabolism to regulate stress resistance and lifespan. **a** Diagram of the kynurenine pathway. 5 mM paraquat stress resistance assay comparing the wild type and FMO-2 OE on empty-vector (EV) and **b** *kmo-1* RNAi, **c** *tdo-2* RNAi, and **d** *nkat-1* RNAi. Lifespan assay comparing the survival of the wild type, FMO-2 OE, and FMO-2 KO on EV, and **e** *kmo-1* RNAi, **f** *tdo-2* RNAi, and **g** *nkat-1* RNAi. Percent change in mean lifespan compared to their respective EV controls are shown in the left bottom corner of the figures. Black circle = wild type, blue diamond = FMO-2 OE, and red triangle = FMO-2 KO. Solid line = EV and dotted line = RNAi. * denotes significant change in mean lifespan at *p* < 0.05 using log-rank and # denotes significant interaction between the RNAi of interest and *fmo-2* genotype at *p* < 0.01 using Cox regression analysis. N.S. = not significant. Statistics are in Supplementary Tables 3, 4, 5 and Supplementary Data 4, 5.

Based on our initial data linking FMO-2 to OCM, it is important to note that in addition to being a key process in the kynurenine pathway, the conversion of tryptophan to N-formylkynurenine precedes the conversion of N-formylkynurenine to kynurenine by formamidase, a process that releases formate, which is also a carbon source for OCM^44^. Formate can enter OCM through the folate cycle, thus providing a connection between tryptophan metabolism, the kynurenine pathway, and OCM. Indeed, we observe a significant interaction between formate supplementation and FMO-2 OE, whereby supplementing the worm diet with formate extends the lifespan of the wild-type without further extending the lifespan of FMO-2 OE (Fig. 4i and Table 1). Based on this knowledge, we hypothesize that the kynurenine pathway is a target of FMO-2 that leads to changes in OCM. To test this hypothesis, we assessed whether genes involved in tryptophan metabolism interact with FMO-2 (Fig. 5a). Like our RNAi analyses of the OCM genes, we observe (1) changes in the stress resistance and lifespan of the wild type, FMO-2 OE, and FMO-2 KO worms following the knockdown of genes involved in the kynurenine pathway and (2) interactions between these genes and FMO-2 using the same parameters that we used for the OCM-related genes (Fig. 5b–g, Tables 1 and Supplementary Table 3). Here, we again observed a separation between the regulation of stress resistance and lifespan under *kmo-1* and *tdo-2* knockdown. Knocking down *kmo-1* increases the stress resistance of the wild type and FMO-2 OE, but shows significant interaction with FMO-2 OE, whereby these worms are less affected by the RNAi compared to their wild type counterparts (Fig. 5b, Supplementary Table 3). Conversely, knocking down *kmo-1* decreases the lifespan of the wild type, FMO-2 OE, and FMO-2 KO (Fig. 5e). However, this knockdown shows significant interaction with FMO-2 KO, whereby these worms are less affected by the RNAi compared to their wild type counterparts (Table 1). It is possible that knocking down *kmo-1* increases flux through KYNU-1, whose gene suppression extends lifespan^20^, thereby reducing FMO-2 OE lifespan. Similar to *atic-1* RNAi, this finding potentiates that knocking down *kynu-1* is in the same functional pathway as overexpressing *fmo-2*. Knocking down *tdo-2* decreases the stress resistance of the wild type and FMO-2 OE but has a significant interaction with FMO-2 OE, whereby these worms are more affected by the RNAi compared to their wild type counterparts (Fig. 5c, Supplementary Table 3). Conversely, knocking down *tdo-2* extends the lifespan of the wild type, FMO-2 OE, and FMO-2 KO (Fig. 5f). *Tdo-2* knockdown was previously reported to extend lifespan by inhibiting tryptophan degradation and thereby improving the regulation of proteotoxicity^19^. We find that *tdo-2* knockdown interacts with both FMO-2 KO and FMO-2 OE to modulate longevity, where both strains show reduced lifespan extension compared to wild-type animals (Table 1). Knocking down *tdo-2*, in addition to increasing tryptophan level^19^, may increase *fmo-2* activity as a compensatory response to metabolize tryptophan, thereby resulting in lifespan extension. Similar to *sams-1*, it is likely that there is a functional overlap between *tdo-2* and *fmo-2* in regulating worm lifespan.

Knocking down *nkat-1* increases the stress resistance of the wild type and FMO-2 OE but does not show a significant interaction with FMO-2 OE, consistent with it not acting in the same functional pathway as FMO-2 OE to regulate stress resistance (Fig. 5d, Supplementary Table 3). Knocking down *nkat-1* only extends the lifespan of FMO-2 OE but does not show an interaction with it below our statistical threshold (Fig. 5g, Table 1). *nkat-1* is involved in the conversion of kynurenine to kynurenic acid, which is a neuromodulator that has effects on behavior and possibly lifespan^45,46^. We hypothesize that either increased kynurenic acid production could limit lifespan slightly and suppressing kynurenic acid synthesis therefore increase FMO-2 OE, or that suppressing kynurenic acid synthesis may lead to increased flux in other parts of the pathway (e.g., NAD) that may also be beneficial for lifespan under the metabolic changes following *fmo-2* overexpression.

Knocking down *afmd-1* yields inconsistent results in our experiments (Supplementary Table 5). In sum, our data support an interaction between multiple genes involved in the kynurenine pathway and FMO-2. Furthermore, our data again separate stress resistance and longevity, and are consistent with the possibility that FMO-2 confers stress resistance and longevity through separable mechanisms.

To prevent egg hatching, our stress resistance and lifespan experiments use 5-fluorodeoxyuridine (FUdR), which is a compound that can interact with a component of one-carbon metabolism, thymidylate synthase (TYMS-1)^47^. We tested whether this drug influences FMO-2 OE lifespan phenotype. We find that FMO-2 OE extends the worm lifespan even in the absence of FUdR (Supplementary Fig. 5), suggesting that FUdR does not have a major effect on FMO-2-mediated lifespan extension and consistent with published data showing that FUdR is also not required for decreased *sams-1* to extend lifespan^48^. While these results suggest that FUdR is not crucial for the effects of FMO-2-mediated lifespan extension, they do not rule out some other interaction within the pathways tested, and should be considered when interpreting the data.

## Discussion

**Fig. 6:**
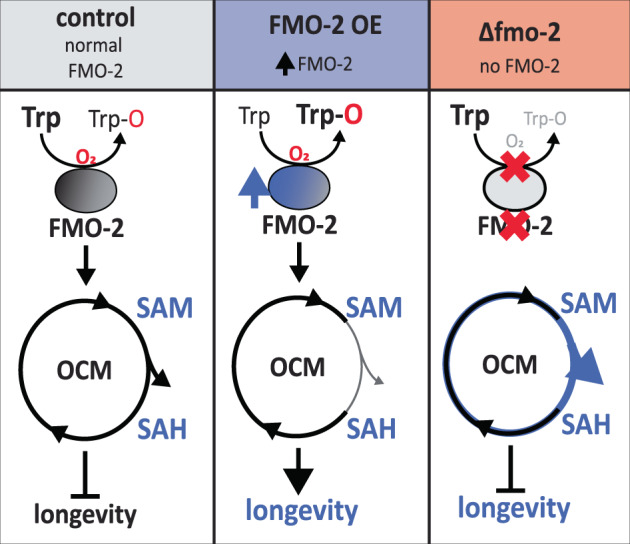
Proposed model. In control conditions, there is very low *fmo-2* expression, leading to low levels of tryptophan metabolism/kynurenine production through FMO-2, and maintaining normal flux through one carbon metabolism and normal lifespan. When *fmo-2* is induced, FMO-2 oxygenates tryptophan, leading to increased kynurenine production and decreased methylation output flux through OCM, thereby extending nematode lifespan. When *fmo-2* is absent, these metabolic changes do not occur, preventing an extension in lifespan. The gray line represents decreased flux and the blue line represents increased flux.

For half a century, FMOs have been primarily classified as xenobiotic enzymes. However, the mechanisms by which these enzymes affect endogenous metabolism are still not well studied, with the exception of human FMO1 (and FMO3) and mouse FMO1 converting hypotaurine to taurine^49^. Based on our data, we propose a model where overexpression of *fmo-2* to levels similar those observed under hypoxia and dietary restriction is sufficient to remodel metabolism in the nematode *C. elegans* (Fig. 6). Here, we show that *fmo-2*, a regulator of longevity that is critical for lifespan extension and stress response under dietary restriction and hypoxia, interacts with both tryptophan and one-carbon metabolism to confer longevity and stress resistance benefits. We find that modulating the expression of a single oxygenating protein can cause a multitude of metabolic and physiological effects, similar to the activation of transcription factors and kinases. Our results suggest a broader, more significant role for FMO-2 than previously known.

Our data are consistent with a model where the reduction of flux through the methylation pathway leads to longevity benefits. By projecting gene expression data to a stoichiometric model for OCM metabolism, we predict that FMO overexpression results in a reduction in methylation flux. This model-based prediction based on gene expression data is experimentally validated, indicating that this approach can be a powerful tool to simplify the understanding of complex metabolic pathways and to study the biology of aging. Perturbation in the SAM/SAH ratio by either the supplementation of metformin or a mutation in *sams-1* also extends worm lifespan^13,17^. While multiple studies report that methionine restriction robustly extends lifespan across species, including worms, flies, and mice^13,50,51^, others show that exogenous supplementation of methionine is not detrimental to lifespan^52^. In our study, we observe a level of methionine that shows an increasing trend with increasing *fmo-2* expression level (Fig. 1d). Although SAM levels depend on methionine, there is precedent for SAM levels being low even when methionine levels are high^53^. Two potential reasons for these observations could be (1) FMO-2 blocks methionine to SAM conversion, which would increase methionine, or (2) there could be an increase in the fluxes of internal reactions within OCM. We hypothesize that #2 is more likely, since methionine is not an in vitro substrate for FMO-2 (Supplementary Table 9). Taken together, these findings suggest that methionine utilization rather than methionine abundance is a key factor that influences the aging process.

Although suppressing *sams-1* expression phenocopies FMO-2 OE lifespan in the wild type and FMO-2 KO, doing so reduces the stress resistance of the worms against paraquat. This separation of lifespan and stress resistance is occasionally observed under other long-lived conditions^54^. It is unclear if simply reducing methylation is sufficient to promote longevity benefits, or if this mechanism requires suppression of specific methylation processes. It will be important for future studies to determine how cells regulate different methylation fluxes under *sams-1* knockdown and decreased overall methylation. One potential mechanism under this genetic condition could be that specific methyltransferases that are essential for survival will have higher affinity to methyl groups to outcompete other nonessential or deleterious methyltransferases. It will also be interesting to test why both FMO-2 OE and *sams-1* RNAi extend lifespan, while causing opposite effects on paraquat resistance. We hypothesize that the reduction of SAM level in *sams-1* RNAi is more severe than in FMO-2 OE, which could cause more severe effects, such as a reduction in stress resistance. This could explain some of the phenotypes observed in *sams-1* RNAi animals (e.g., delayed development^48^) that we do not observe in FMO-2 OE^4^.

We note that while our data suggest methylation as the key downstream effector of FMO-2, we have not excluded the possibility that the transsulfuration pathway may also be involved in this mechanism. The transsulfuration pathway is reported to be a necessary and sufficient component of DR-mediated lifespan extension in flies^16^. It will be interesting to determine the mechanistic relationship between the transsulfuration and transmethylation pathways in regulating longevity. We also note that the stress resistance and lifespan experiments use FUdR. FUdR is a potential confounding influence, and although FMO-2 OE mediated lifespan extension is independent of FUdR, its effect cannot be fully excluded since it wasn’t studied in all of the conditions. We also note that we considered whether our findings could result from an artifact of an overexpression model of FMO-2. However, we believe this is unlikely based on our previous work establishing FMO-2 as a regulator of longevity downstream of DR^4^ and a more recently published work reporting that OCM is altered by the DR genetic model *eat-2* mutant^14^. Similarly, we note that these experiments use RNAi, and that while some of these enzymes are essential and null mutants are not available, others are available and could prove useful in future studies focusing on both OCM and the tryptophan pathway.

Our data also support an interaction between *fmo-2* and tryptophan metabolism to influence longevity. These findings are particularly interesting because we identify a putative endogenous metabolic pathway of FMOs in relation to the aging process. Based on cell line metabolomics, enzyme kinetics, and LC-MS data, there are at least two plausible mechanisms for how oxygenation of tryptophan by FMO-2 can lead to the synthesis of N-formylkynurenine. First, FMOs across taxa are known to dimerize and form higher-order oligomers^55,56^. Therefore, it is possible that FMO-2 dimerizes and dioxygenates tryptophan forming N-formyl-kynurenine, which is then converted to kynurenine by formamidase. Second, the same process could be involved in subsequent oxygenation by FMO-2 monomers, but it is unknown how stable a monooxygenated form of tryptophan would be within the cell, making the first mechanism more likely. To our knowledge this is a primary example of the dioxygenation of a substrate that could potentially require dimerized FMOs. The mechanism of this reaction and its potential requirement of dimerized FMOs will be a target of future research. Furthermore, the dioxygenation of tryptophan by FMOs is especially interesting considering only dioxygenases, such as *tdo-2*, *ido-1*, and *ido-2*, have been shown to mediate the conversion of tryptophan to N-formylkynurenine^19^. Regardless, our data implicate tryptophan as a bona fide in vitro and likely in vivo substrate of animal FMOs either through dioxygenating or monooxygenating mechanisms. Although we tested multiple conventional and unconventional FMO substrates, such as methimazole^55–57^ and 2-heptanone^58^ (Supplementary Table 9), respectively, much work remains to fully establish the general FMO-2 substrate profile and how it compares to those of mammalian FMOs. We note that the pathway analysis (Supplementary Table 1) also examined tryptophan metabolism, but as a pathway it was not significantly enriched. This could be due to one of at least 3 reasons: (1) while there are 30 total metabolites within the tryptophan pathway, the untargeted metabolomics assay was only able to cleanly identify 5 of the 30 metabolites, two of which were significantly altered. Many metabolites within this pathway are transient or present at very low concentrations, requiring more specialized sample preparation or additional targeted metabolomics methods to measure directly. Using our general untargeted metabolomics method we were therefore unable to measure much of the pathway. (2) It is possible that FMO-2’s effects on tryptophan lead to compensatory responses elsewhere in the tryptophan pathway that mask the effects, or (3) FMO-2 may also metabolize tryptophan to compounds other than NFK that do not lead to changes throughout the canonical tryptophan pathway. We favor explanation #1, as the untargeted data were always meant to provide clues for further analysis and do not have the same scope and statistical power for every pathway. Unlike the pathway analysis, which considers multiple metabolites for statistics, our initial identification of tryptophan metabolism was based on a change of a single metabolite, tryptophan.

Our data support a model where the interaction between FMO-2 and tryptophan metabolism directly or indirectly modulates the metabolite profile of OCM, altering flux patterns that are consistent with our computational model predictions and subsequent genetic analyses. Further investigation is needed to understand the complete details of the *fmo-2*-mediated connection between OCM and tryptophan in regulating lifespan. Based on the knowledge we gained from this study and previous work, we propose the following two mechanisms may occur together or separately: (1) Oxygenation of tryptophan by FMO-2 alters OCM flux by increasing formate levels as a direct link between tryptophan metabolism and OCM. Formate is a single carbon-containing molecule that can enter the folate cycle as a carbon source^44^, and we find that formate addition extends WT lifespan without affecting FMO-2 OE. Formate is generated as a byproduct when kynurenine is synthesized from N-formylkynurenine by formamidase^44^. It is possible that increased formate levels are causal in conferring stress resistance and longevity benefits under metabolically stressful conditions, such as DR or hypoxia. This hypothesis could explain why knocking down *alh-3* reduces FMO-2 OE lifespan. Knockdown of *alh-3* would prevent the breakdown of 10-formyl-THF and synthesis of THF, which would then prevent formate from entering OCM by being converted into 10-formyl-THF^59–61^. Since we hypothesize that increased formate enters OCM to affect worm lifespan, preventing the incorporation of formate into OCM would result in an accumulation of formate, a molecule that has been reported to be toxic in human cells^62^. Thus, this would result in the reduction of lifespan specifically for FMO-2 OE, as we have observed. (2) FMO-2 interacts with the mechanistic target of rapamycin (mTOR). Dietary restriction leads to inhibition of mTOR signaling, which is a central regulator of lifespan and aging^63^. Interestingly, both DR- and rapamycin-mediated mTOR inhibition induce the expression of FMOs. A recent study shows that diaminodiphenyl sulfone (DDS) induces the expression of *fmo-2* and extends lifespan, but it does not further extend lifespan in combination with rapamycin^41^. This finding is consistent with the hypothesis that *fmo-2* interacts with mTOR inhibition to extend lifespan. We also show that *fmo-2* interacts with SAM and tryptophan metabolism, both of which are known to alter mTOR activity^64–66^. Thus, examination into the role of mTOR in *fmo-*2-mediated lifespan extension is warranted.

Taken together, our study expands the role of FMO-2 from a xenobiotic enzyme to a metabolic regulator of longevity that has global effects on the metabolome in worms. In particular, the identification of OCM as a target of FMO-2 has implications outside the aging field, considering that OCM remodeling has been studied under the context of cancer biology for more than 70 years^67^. Furthermore, through the identification of tryptophan as a putative substrate for CeFMO-2, this study highlights the conserved importance of FMOs in multiple contexts, including aging and many diseases where OCM and/or the kynurenine pathway play a role. These findings illustrate the potential for therapeutic targets of these proteins for treating age-related diseases and/or increasing longevity and healthspan. This exciting translational potential for the conserved roles of FMOs will be a target for future research.

## Methods

Ethical approval or guidance was not required as local laws governing the use of research animals do not apply to invertebrate models.

### Strains and growth conditions

Standard *C. elegans* cultivation procedures were used as previously described^4,68^. Briefly, N2 wild type, KAE9 ((*eft-3p*::*fmo-2* + *h2b*::*gfp* + Cbr-*unc-119*(+)), and VC1668 (*fmo-2(ok2147*)) strains were maintained on solid nematode growth media (NGM) using *E. coli* OP50 throughout life except where RNAi (*E. coli* HT115) were used. Worms were transferred or picked gently using a platinum wire. All experiments were conducted at 20 °C.

### Metabolomics

OP50 bacteria were treated with 0.5% (v/v) paraformaldehyde (50980495, Fisher Scientific) as previously described^68^. Briefly, overnight bacterial culture was treated with 0.5% paraformaldehyde (PFA) and incubated at 37 °C in a shaking incubator at 200 rpm for 1 h. The treated bacteria were centrifuged and washed with LB five times to remove residual PFA before seeding onto 100 mm NGM plates. Approximately 500 eggs were put on these plates and grown until they reached late L4 larval stage. The worms were washed off the plates with 10 mL of M9 buffer and were collected in 15 mL conical tubes. The worms were pelleted using a clinical centrifuge for 1 min at 150 × *g* and the supernatant was vacuum aspirated. The worms were washed once with 10 mL of M9 buffer and then with 10 mL of 150 mM ammonium acetate (A637, Fisher Scientific) to remove phosphates from M9, each time being centrifuged and the supernatant being aspirated. After these washing steps, the pellets were flash frozen in liquid nitrogen.

Metabolites were extracted from pellets by addition of 500 µL of ice-cold 9:1 methanol: chloroform, followed immediately by probe sonication for 30 s with a Branson 450 Sonicator. The resulting homogenates were kept on ice for 5 min and were then centrifuged for 10 min at 4000 × *g* at 4 °C. Supernatant was then transferred to autosampler vials for analysis. Hydrophilic interaction liquid chromatography-electrospray ionization mass spectrometry (HILIC-LC-ESI-MS) analysis was performed in negative ion mode using an Agilent 1200 LC system coupled to an Agilent 6220 time-of-flight mass spectrometer equipped with a Dual ESI source. Chromatography was performed as previously described^69,70^. Briefly, a Phenomenex Luna NH2 column was used with dimensions of 150 mm × 1.0 mm i.d., the flow rate was 0.07 mL/min, and the injection volume was 10 µL. Mobile phase composition was as follows: mobile phase A was acetonitrile, and mobile phase B was 5 mM ammonium acetate in water adjusted to pH 9.9 using ammonium hydroxide. The gradient was used as follows: 20% to 100% B over 15 min, 3 min hold at 100% B, then return to 20% B at 18.1 min and re-equilibrate for 12 min. MS source and data acquisition parameters were as follows: drying gas temperature 350 °C, drying gas flow 10 L/min, nebulizer pressure 20 psi, capillary voltage 3500 V, fragmentor voltage 175 V, MS scan range 50–1200 *m*/*z*, scan rate 1 spectrum/s, reference mass correction enabled using 1.25 µM HP-0921 reference compound (Agilent, Santa Clara, CA). Untargeted peak detection and alignment was performed using XCMS^71^ using the following parameters: peak finding method: centWave, maximum ppm deviation: 30, min/max peak width: 10/60 s, signal/noise threshold: 6, minimum *m*/*z* difference for peaks with overlapping retention time: 0.01, alignment bandwidth 10 s *m*/*z* slice width for peak grouping 0.025; remaining parameters were set at default values.

The resulting untargeted metabolomics data were analyzed using Metaboanalyst 4.0 (http://metaboanalyst.ca). Within Metaboanalyst, the data were median normalized, adjusted using auto scaling, and were then subjected to principal component analysis using default parameters. Pathway analysis was performed using Metaboanalyst’s functional analysis module, which is an enhanced implementation of the original Mummichog pathway and network analysis algorithm. *P*-values and t-scores of each MS peak data were calculated between the wild type and FMO-2 OE (Supplementary Data 2). Mass tolerance was set to 10 parts per million (ppm) and mummichog algorithm *p*-value cutoff was set to 0.05. Default parameters were used for other settings and the analysis was done using the *C. elegans* pathway library. The mummichog algorithm produces putative compound annotation for interpretation at the pathway level. For individual features of interest, compound identities assigned by the algorithm were validated by targeted analysis using authentic standards as described below.

Targeted metabolomics analysis used the same LC-MS parameters as untargeted, but data analysis was performed using Agilent MassHunter Quantitative Analysis software. Metabolite identification was performed by matching accurate mass and retention time with authentic standards analyzed in-house on the same method. Statistical analysis for targeted metabolomics data was done using Microsoft Excel (Microsoft 365) following median normalization and log transformation.

### Stress resistance assay

Paraquat (Methyl viologen dichloride hydrate, 856177, Sigma-Aldrich) was used to induce oxidative stress. Worms were synchronized from eggs on RNAi plates seeded with *E. coli* HT115 strain expressing dsRNAi for a particular gene and at L4 stage 40 worms were transferred on RNAi-FUdR (40690016, Bioworld) plates containing 5 mM paraquat. A minimum of two plates per strain per condition were used per replicate experiment. As described previously, worms were then scored every day and considered dead when they did not move in response to prodding under a dissection microscope^4^. Worms that crawled off the plate were not considered, but ruptured worms were noted and considered.

### Lifespans

Gravid adults were placed on NGM plates containing 1 mM β-D-isothiogalactopyranoside (367931, Fisher Scientific), 25 μg/ml carbenicillin (BP26485, Fisher Scientific), and the corresponding RNAi clone from the Vidal or Ahringer RNAi library. After 3 h, the adults were removed, and the eggs were allowed to develop at 20 °C until they reached late L4/young adult stage. From here, 40 to 90 worms were placed on each RNAi plate and transferred to fresh RNAi + FUdR plates on day 1, day 2, day 4, and day 6 of adulthood. A minimum of two plates per strain per condition were used per replicate experiment. Experimental animals were scored every 2–3 days and considered dead when they did not move in response to prodding under a dissection microscope. Worms that crawled off the plate were not considered, but ruptured worms were considered. A similar method was used for formate (F0507, Sigma-Aldrich) and s-adenosylmethionine (PureBulk, Roseburg, OR, USA) supplementation lifespan experiments, except either 1 mM formate or 2 mM s-adenosylmethionine was added to the NGM plates without IPTG. A similar method was also used for the non-FUdR lifespan experiments, except FUdR was not added to the plates and worms were fed *E. coli* OP50.

### Cox regression methods

Supplementary Data 4 and 5 provide the results of Cox proportional hazards regression models, which were run in Stata 14. The model includes a categorical variable for strain, using Wild Type (N2) as the base category, and including dummy variables for FMO-2 OE and/or FMO-2 KO. It also includes a dummy variable for the individual RNAi versus empty vector (EV) control. Variables of particular interest for this paper are the interactions between the RNAi dummy and the *fmo-2* mutant dummies, which capture the differential effect and interaction of various RNAi on *fmo-2* mutants versus control worms. To account for multiple testing, only interactions with a *p*-value <0.01 were considered to be significant.

### Computational modeling

The computer model was generated by building a stoichiometric matrix S (10 reactants by 13 reactions), accounting for all reactions shown in Fig. 4a. A steady-state approximation was used, as shown in Eq. 1. In Eq. 1, S is the stoichiometric matrix and **J** is a vector of fluxes for each of the reactions.

1 $${{{{{\rm{S}}}}}}\cdot {{{{{\bf{J}}}}}}={{{{{\bf{0}}}}}}$$

To obtain a biologically relevant solution, we projected the expression data of genes involved in the reactions used in the model to the nullspace of S by solving for Eq. 2. Single genes were used as representative genes for each reaction to simplify the model. Gene expressions related to input fluxes were assumed to be one for all strains. Reactions used in the model and the relevant gene expression data are shown in Supplementary Tables 6 and 7. In Eq. 2, M is the nullspace of S, **b** is the vector of relative gene expression data from the wild type, FMO-2 OE or FMO-2 KO that have been normalized to the wild type, and **x** is a vector such that S**x** is the projection of **b** onto the column space of M, which gives us the vector of reaction fluxes, **J**, within the nullspace of S. To account for data variability, expression level with greater than 0.5x or less than 1.5x fold changes were assumed to be equal to the wild type control. Equation 2 was solved using the lsqminnorm function in MATLAB 2018a. The lsqminnorm function returns the minimum norm least-squares solution to M**x** = **b** by minimizing both the norm of M * **x** – **b** and the norm of **x**.

2 $${{{{{\rm{M}}}}}}\cdot {{{{{\bf{x}}}}}}={{{{{\bf{b}}}}}}$$

The inner product of the resulting vector **x** and the nullspace matrix M was obtained to calculate the reaction flux predictions resulting from the gene expression projection as shown in Eq. 3. The calculated **J** for FMO-2 OE and FMO-2 KO were normalized to that of the wild type to obtain the relative fluxes.

3 $${{{{{\rm{M}}}}}}\cdot {{{{{\bf{x}}}}}}={{{{{\bf{J}}}}}}$$

### Quantitative PCR

RNA was isolated from day 1 adult worms following three rounds of freeze-thaw in liquid nitrogen using Invitrogen’s Trizol extraction method and 1 µg of RNA was reverse transcribed to cDNA using SuperScript™ II Reverse Transcriptase (18064071, Invitrogen,). Gene expression levels were measured using 1 μg of cDNA and SYBR^TM^ Green PCR Mastermix (A25742, Applied Biosystems) and primers at 10 μM concentration. mRNA levels were normalized using previously published housekeeping gene controls, *tba-1* and *pmp-3*^72^. For the RNAi validation, same protocol was used for RNA isolation and cDNA preparation, and Y45F10D.4 was used as a reference gene^73^. List of primers used are in Supplementary Table 12.

### Enzyme kinetic assays

Oxygenation activity of FMO-2 was characterized using the method previously described^74^. Briefly, oxygenation of substrates was determined by spectrophotometrically following the consumption of NADPH at 340 nm using the molar extinction coefficient 6.22 mM^−1^ cm^−1^. Components of the assay buffer included 25 mM sodium phosphate buffer (pH 8.5), 0.5 mM diethylenetriaminepentaacetic acid (DETAPAC), 0.5 mM NADPH, and 0.04 µM FMO-2 with excess FAD. The final substrate concentrations for tryptophan were 100, 250, 500, 750 µM and 1, 2.5, 5, 7.5, and 10 mM. The final substrate concentrations for MMI were 100, 300, and 600 µM and 1, 3, 5, 7, 10, and 30 mM. To determine the rate of oxidation of NADPH by FMO, NADPH concentrations of 10, 30, 100, 300, 500, and 700 µM and 1 and 1.5 mM were used. Experiments were conducted at 30 °C while shaking. Kinetic parameters (i.e., *k*_*cat*_ and *K*_*m*_) were determined by fitting plots of the rate of turnover vs the substrate concentration to the Michaelis-Menten equation using GraphPad Prism (version 9.1.0; GraphPad Software Inc., San Diego, CA). Purified FMO-2 protein was purchased from GenScript. NADPH (10107824001), FAD (F6625), MMI, L-tryptophan (T0254), and all other substrates were purchased from Sigma-Aldrich (St. Louis, MO). DETAPAC (AC114322500) and sodium phosphate buffer (S374-500) were purchased from Fisher (Waltham, MA).

### In vitro studies LC-MS

Analysis of samples from in vitro studies with purified FMO2 was performed using LC-MS with untargeted feature detection. Samples contained 100, 250, or 500 µM tryptophan in the same conditions as the enzymatic assays with FMO-2 protein. 100 µL of conditioned media were vortexed with 400 µL of 1:1:1 methanol:acetonitrile:acetone to precipitate protein. The extract was centrifuged for 10 min at 16,000 × *g* and 200 µL of supernatant were transferred to a clean autosampler vial with insert and dried under a stream of nitrogen gas. The dried extract was reconstituted in 50 µL of 85/15 acetonitrile/water and analyzed by HILIC-TOF-MS on an Agilent 1290 Infinity II/Agilent 6545 QTOF. Chromatography was performed on a Waters BEH Amide column (2.1 mm ID × 10 cm, 1.7 µm particle diameter) with mobile phase prepared as described previously^75^ except that mobile phase A contained 5% acetonitrile. Briefly, the flow rate was 0.3 mL/min, the column temperature 55 °C, and the gradient was as follows: 0–0.70 min 100%B, 0.7–6.7 min 100–85%B, 6.7–8.7 min 85%B, 8.7–16 min 85–28%B, 16–16.7 min 28%B, 16.7–16.8, 28–0%B. Total run time was 22 min. Ion polarity was positive, gas temp was 320 °C, drying gas was 8 L/min, nebulizer was 35 psi, sheath gas temp and flow were 350 °C and 11 L/min, capillary voltage 3500 V. The instrument was operated in full scan mode at 2 spectra/s and a mass range of 50–1200 Da. Feature detection and alignment were performed using XCMS (3.16). Potential reaction products were detected by computationally examining the data for features present in each sample set. Identification of potential reaction products was performed using MS/MS data acquired from a pooled sample.

### *Fmo-2* induction experiment

Synchronized L4 *fmo-2p::mCherry* transcriptional reporter strain animals were incubated overnight in S-media enriched with OP50 with and without 1 mM tryptophan in a 96-well plate. Some worms were also incubated overnight in S-media without OP50 as a positive control to induce *fmo-2* expression under dietary restriction.

### Microscopy

Images were acquired with the LASx software and Leica scope at ×6.3 magnification with more than 15 worms per treatment. Prior to imaging, worms were anesthetized with 0.5 M sodium azide (NaN3) on a 2% agarose pad. Fluorescence mean comparisons were calculated using ImageJ software.

### Statistical analyses

Log-rank test was used to derive *p*-value for lifespan and paraquat survival assays using *p* < 0.05 cut-off threshold compared to EV controls via OASIS 2^76^. Unpaired Student’s t-test was used to derive *p*-values for targeted metabolomics data using *p* < 0.05 cut-off threshold compared to the wild type. Using one-way ANOVA for trend analysis, *p*-values were calculated for *fmo-2* expression levels that affect OCM metabolite levels. Unpaired Student’s t-test was used to derive *p*-values for comparing the metabolomics data of HepG2 pDEST control and FMO2, FMO4, and FMO5 OE cell lines using *p* < 0.05 cut-off threshold.

### Reporting summary

Further information on research design is available in the Nature Portfolio Reporting Summary linked to this article.

## Supplementary information

Supplementary Information

Description of Additional Supplementary Files

Supplementary Data 1

Supplementary Data 2

Supplementary Data 3

Supplementary Data 4

Supplementary Data 5

Supplementary Data 6

Supplementary Data 7

Reporting Summary

## Data Availability

All data are available in the supplementary files, Supplementary tables, and source data file. All data will also be available upon request. Source data are provided with this paper.

## Source data

Source Data

## Code availability

All MATLAB codes generated in this manuscript are available in Supplementary data 6.
