# Supplementary material for: FMO rewires metabolism to promote longevity through tryptophan and one carbon metabolism in C. elegans
Source: Nat Commun. 2023 Feb 2;14:562. doi: 10.1038/s41467-023-36181-0 (PMC9894935; doi:10.1038/s41467-023-36181-0)
Supplement: Supplementary file 1 — Supplementary Information [file 41467_2023_36181_MOESM1_ESM.pdf]

SUPPLEMENTARY INFORMATION

FMO rewires metabolism to promote longevity through tryptophan and one carbon metabolism in C. elegans

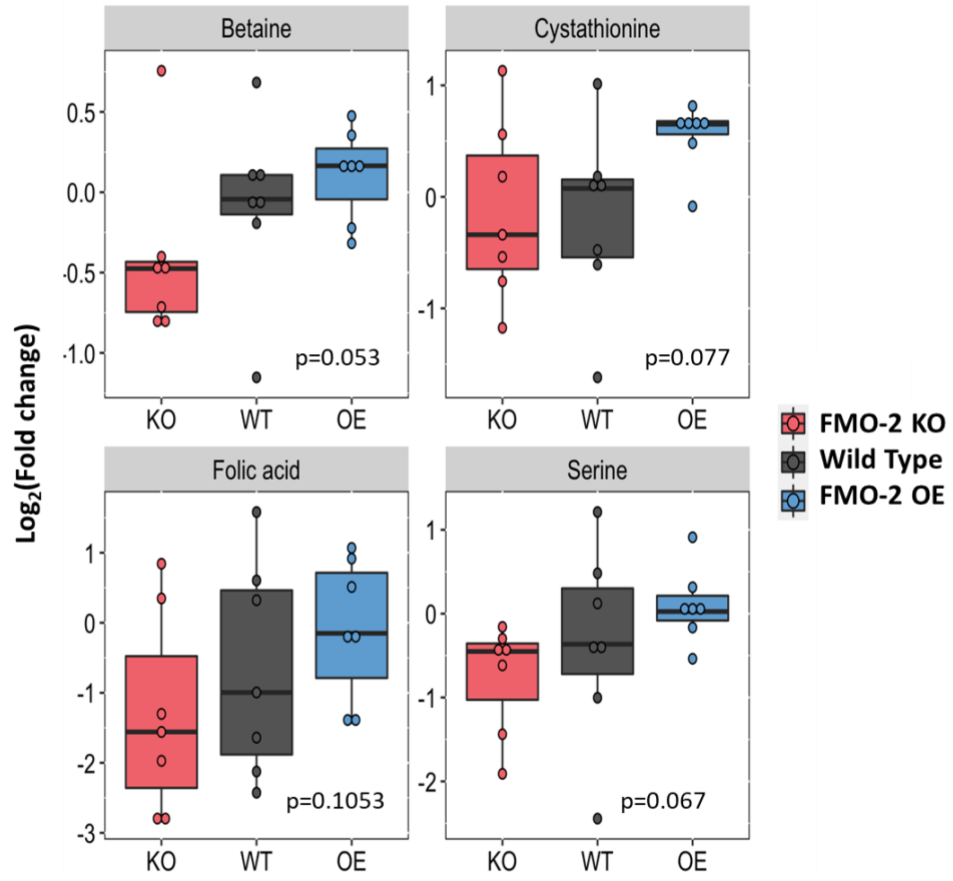

Supplementary Figure 1: Comparison of targeted metabolomics data of metabolites related to OCM between the wild type, FMO-2 OE and FMO-2 KO. p-values are derived from one-way ANOVA trend analysis. Statistics are in Supplementary Table 2. No notes = Not significant. n=7 biologically independent experiments. In box plots, the median is shown by the center line. The box plot represents the normalized intensities of metabolites from wild type (black), FMO-2 OE (blue) and FMO-2 KO (red). The upper boundary of the box represents the 75% interquartile range, while the lower boundary represents the 25% interquartile range.

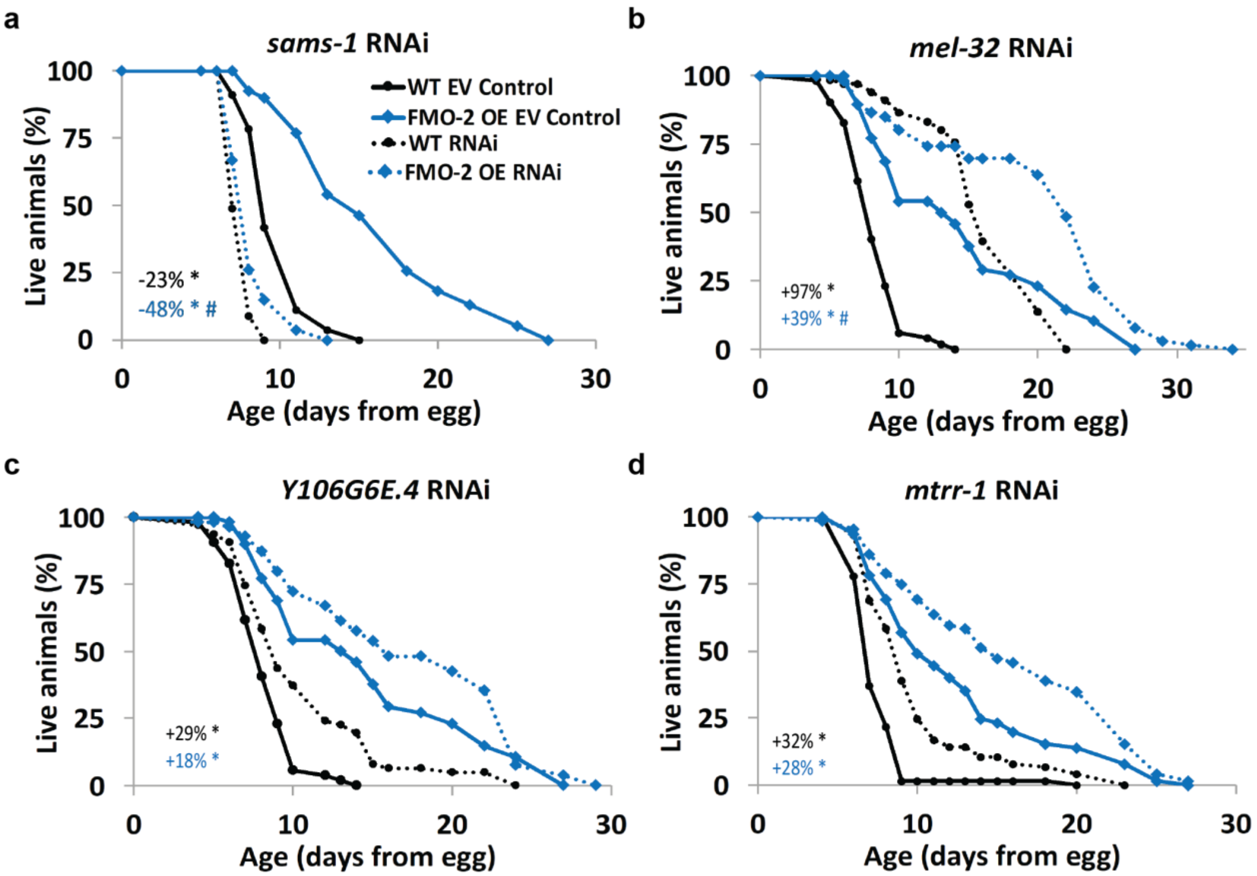

Supplementary Figure 2: *Fmo-2* interacts with OCM genes to regulate stress resistance. 5 mM paraquat stress resistance assay comparing the wild type and FMO-2 OE on empty-vector (EV) and A) *sams-1* RNAi, B) *mel-32* RNAi, C) *Y106G6E.4* RNAi, and D) *mtrr-1* RNAi. Percent change in mean survival compared to their respective EV controls are shown in the left bottom corner of the figures. Black circle = wild type and blue diamond = FMO-2 OE. Solid line = EV, dotted line = RNAi. * denotes significant change in mean survival at $p < 0.05$ using log-rank. # denotes significant interaction with the RNAi at $p < 0.01$ using Cox regression. Statistics are in Supplemental Table 4 and Supplementary Data 4.

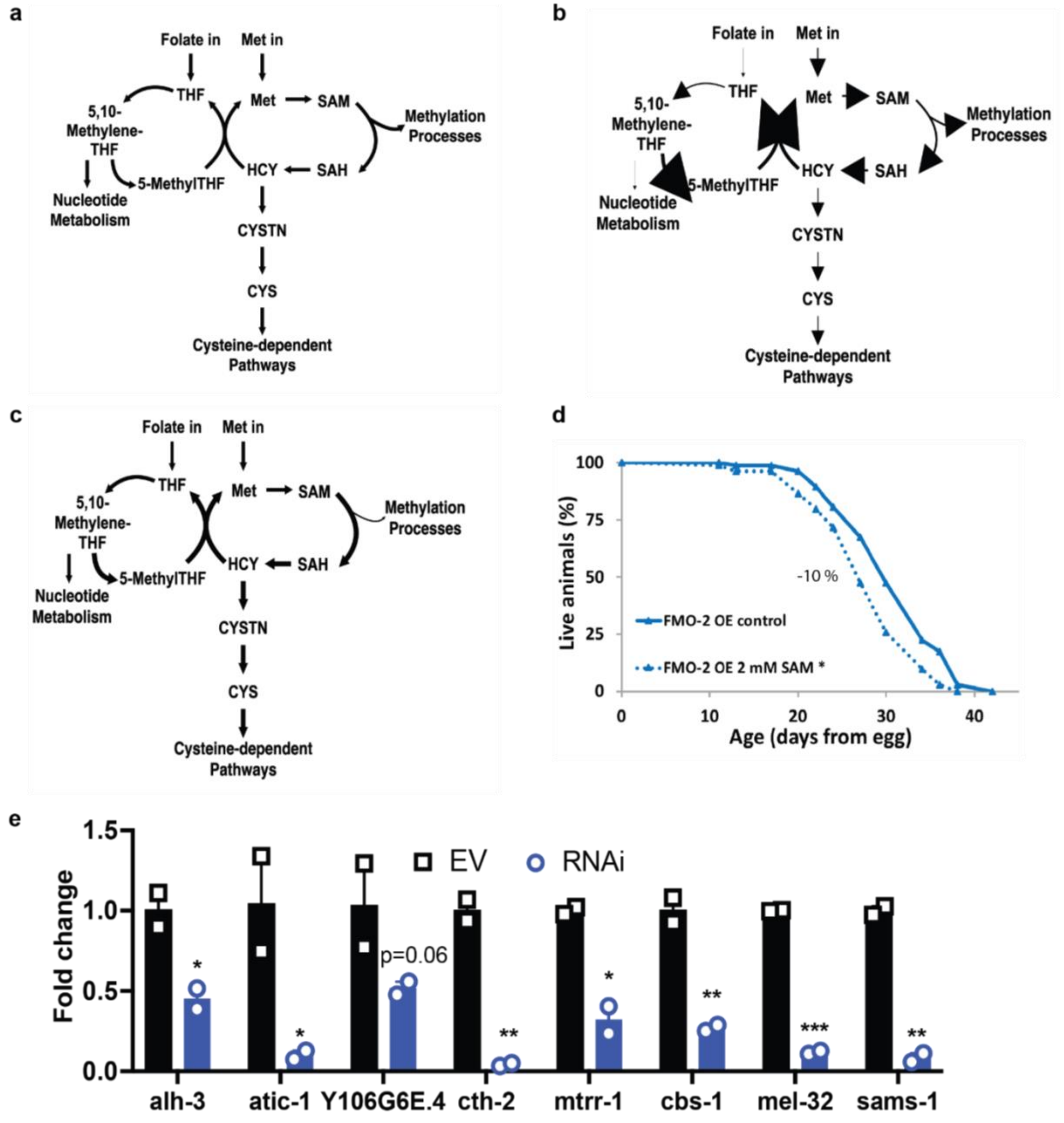

Supplementary Figure 3: Computational model predicts reduced flux through methylation processes. Model predictions of OCM fluxes for A) the wild type, B) FMO-2 OE, and C) FMO-2 KO after normalization to the wild type. Arrow weights represent changes in each flux relative to the wild type, which is set to be equal to 1. D) Lifespan assay comparing the survival of FMO-2 OE on control (solid line) and 2 mM s-adenosylmethionine supplementation conditions (dotted line). * denotes significant change in mean lifespan at $p < 0.05$ using log-rank. E) Validation of

RNAi-mediated knockdown by qPCR. Black color bar = EV (Empty vector), blue color bar = RNAi. p-value = 0.022 (EV vs alh-3), 0.014 (EV vs atic-1), 0.066 (EV vs Y106G6E.4), 0.0025 (EV vs cth-2), 0.024 (EV vs mtrr-1), 0.0031(EV vs cbs-1), 0.00073 (EV vs mel-32), 0.008 (EV vs sams-1). *n = 2* biologically independent experiments. Data are represented as mean values +/- SEM, and the *, ** and *** represent p-values of less than 0.05, 0.01 and 0.001, respectively, using one-tailed unpaired students t-tests. Statistics are in Supplemental Table 8.

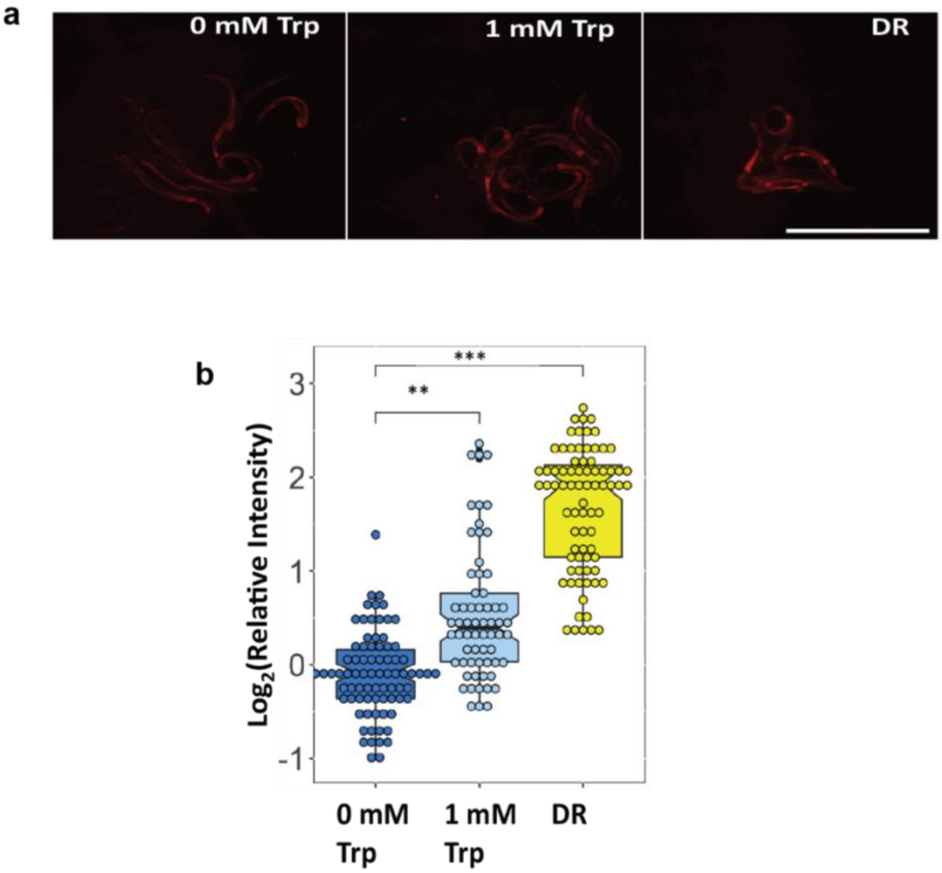

Supplementary Figure 4: Tryptophan induces *fmo-2*. Fluorescence images (A) and quantification (B) of *fmo-2p::mCherry* transcriptional reporter strain under 0 mM tryptophan (fed, negative control), 1 mM tryptophan supplementation and dietary restriction (positive control) conditions. Scale bar = 1 mm. Dark blue color = 0 mM Trp, light blue color = 1 mM Trp, and yellow color = DR. n = 78 (0 mM Trp), 63 (1 mM Trp), 77 (DR) biologically independent animals from three independent experiments. p-value = 0.007 (0mM vs 1 mM Trp), 7.77e-15 (0mM vs DR). ** and *** denotes p-value < 0.01 and <0.0001, respectively, using one-way ANOVA with Tukey's multiple comparison test. The center line of the box represents the median. The upper boundary of the box represents the 75% interquartile range, while the lower boundary represents the 25% interquartile range.

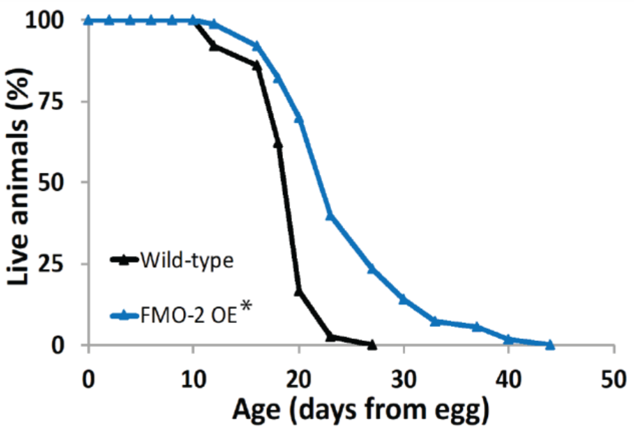

Supplementary Figure 5: FMO-2 extends worm lifespan in the absence of FUdR. Lifespan assay comparing the survival of the wild type (black) and FMO-2 OE (blue) in the absence of FUdR. * denotes significant change in mean lifespan at $p < 0.05$ using log-rank. Statistics are in Supplementary Table 11.

Supplementary Table 1: Pathway enrichment analysis of metabolites in the wild-type and FMO-2 OE

Wild-type vs FMO-2 OE	Path way total	Hits. total	Hits. sig	Expect ed	FET (p-value)	EASE	Gam ma	Emp .Hits	Empiri cal	Pathway Number
Glycine, serine and threonine metabolism	25	13	13	3.183	8.70E-05	0.001	0.003	0	0	P1
Cysteine and methionine metabolism	34	10	9	4.329	0.010	0.049	0.004	0	0	P2
beta-Alanine metabolism	13	7	7	1.655	0.007	0.058	0.004	0	0	P3
Aminoacyl-tRNA biosynthesis	20	14	11	2.546	0.026	0.079	0.004	0	0	P4
Valine, leucine and isoleucine biosynthesis	8	5	5	1.019	0.030	0.181	0.006	0	0	P5
Valine, leucine and isoleucine degradation	35	16	11	4.456	0.099	0.212	0.006	0	0	P6
Alanine, aspartate and glutamate metabolism	20	11	8	2.546	0.110	0.263	0.007	2	0.02	P7
Propanoate metabolism	19	8	6	2.419	0.143	0.355	0.009	7	0.07	P8
Amino sugar and nucleotide sugar metabolism	27	9	6	3.438	0.254	0.494	0.013	10	0.1	P9
Butanoate metabolism	12	7	5	1.528	0.227	0.495	0.013	0	0	P10
Pantothenate and CoA biosynthesis	16	5	4	2.037	0.187	0.495	0.013	5	0.05	P11
Inositol phosphate metabolism	17	3	3	2.164	0.125	0.496	0.013	0	0	P12
Fructose and mannose metabolism	16	4	3	2.037	0.314	0.686	0.025	0	0	P13
Sphingolipid metabolism	9	4	3	1.146	0.314	0.686	0.025	6	0.06	P14
Terpenoid backbone biosynthesis	12	4	3	1.528	0.314	0.686	0.025	0	0	P15
Glyoxylate and dicarboxylate metabolism	30	11	6	3.820	0.507	0.726	0.029	9	0.09	P16
Synthesis and degradation of ketone bodies	4	2	2	0.509	0.251	0.749	0.032	0	0	P17

D-Arginine and D-ornithine metabolism	4	2	2	0.509	0.251	0.749	0.032	0	0	P18
N-Glycan biosynthesis	35	2	2	4.456	0.251	0.749	0.032	0	0	P19
Mucin type O-glycan biosynthesis	4	2	2	0.509	0.251	0.749	0.032	0	0	P20
Galactose metabolism	15	5	3	1.910	0.505	0.813	0.042	38	0.38	P21
Arginine and proline metabolism	27	15	7	3.438	0.710	0.854	0.051	6	0.06	P22
Pentose phosphate pathway	21	3	2	2.674	0.504	0.875	0.056	35	0.35	P23
Histidine metabolism	8	3	2	1.019	0.504	0.875	0.056	0	0	P24
Glycerophospholipid metabolism	12	3	2	1.528	0.504	0.875	0.056	4	0.04	P25
Glycolysis / Gluconeogenesis	22	6	3	2.801	0.663	0.892	0.062	39	0.39	P26
Pyruvate metabolism	16	6	3	2.037	0.663	0.892	0.062	39	0.39	P27
Pyrimidine metabolism	40	21	9	5.093	0.828	0.913	0.071	52	0.52	P28
Sulfur metabolism	11	4	2	1.401	0.693	0.938	0.085	18	0.18	P29
Tyrosine metabolism	21	5	2	2.674	0.819	0.970	0.115	29	0.29	P30
Tryptophan metabolism	30	5	2	3.820	0.819	0.970	0.115	0	0	P31
D-Glutamine and D-glutamate metabolism	5	5	2	0.637	0.819	0.970	0.115	0	0	P32
Nicotinate and nicotinamide metabolism	14	5	2	1.782	0.819	0.970	0.115	5	0.05	P33
Glutathione metabolism	17	9	3	2.164	0.917	0.982	0.137	5	0.05	P34
Citrate cycle (TCA cycle)	16	6	2	2.037	0.896	0.985	0.145	29	0.29	P35
Folate biosynthesis	22	6	2	2.801	0.896	0.985	0.145	23	0.23	P36
Purine metabolism	60	23	7	7.639	0.988	0.996	0.203	72	0.72	P37
Pentose and glucuronate interconversions	14	6	1	1.782	0.986	1.000	1.000	39	0.39	P38
Ascorbate and aldarate metabolism	6	4	1	0.764	0.941	1.000	1.000	59	0.59	P39

Fatty acid biosynthesis	6	2	1	0.764	0.754	1.000	1.000	0	0	P40
Fatty acid elongation	29	1	1	3.692	0.502	1.000	1.000	0	0	P41
Fatty acid degradation	35	2	1	4.456	0.754	1.000	1.000	0	0	P42
Arginine biosynthesis	6	3	1	0.764	0.879	1.000	1.000	0	0	P43
Lysine degradation	15	2	1	1.910	0.754	1.000	1.000	0	0	P44
Selenocompound metabolism	14	1	1	1.782	0.502	1.000	1.000	21	0.21	P45
Starch and sucrose metabolism	10	5	1	1.273	0.971	1.000	1.000	39	0.39	P46
Mannose type O-glycan biosynthesis	9	1	1	1.146	0.502	1.000	1.000	0	0	P47
Glycerolipid metabolism	7	1	1	0.891	0.502	1.000	1.000	35	0.35	P48
Arachidonic acid metabolism	12	4	1	1.528	0.941	1.000	1.000	16	0.16	P49
One carbon pool by folate	8	2	1	1.019	0.754	1.000	1.000	23	0.23	P50
Porphyrin and chlorophyll metabolism	17	2	1	2.164	0.754	1.000	1.000	13	0.13	P51
Nitrogen metabolism	5	2	1	0.637	0.754	1.000	1.000	0	0	P52
Drug metabolism - cytochrome P450	13	3	1	1.655	0.879	1.000	1.000	0	0	P53
Drug metabolism - other enzymes	25	5	1	3.183	0.971	1.000	1.000	0	0	P54
Phosphatidylinositol signaling system	15	1	1	1.910	0.502	1.000	1.000	0	0	P55

FET = Fisher’s exact test
EASE = Expression Analysis Systematic Explorer
p-value is calculated using two-tailed Fisher’s exact test

The analysis was done using the untargeted metabolomics data from Supplementary Data 1. Statistics used for the pathway analysis is in Supplementary Data 2.

Supplementary Table 2: Statistics for targeted metabolomics analysis of wild-type, FMO-2 OE, and FMO-2 KO

**Metabolite**	**n**	**Fold change**			**Unpaired t-test (p-value)***			**ANOVA: Test for Trend**
		WT	OE	KO	WT vs OE	OE vs KO	WT vs KO	p-value
Biotin	7	1	0.844	0.768	0.498	0.633	0.258	0.613
Pantothenic acid	7	1	0.831	0.832	0.367	0.897	0.365	0.914
Nicotinic acid	7	1	0.909	1.200	0.994	0.174	0.354	0.317
Choline	7	1	1.592	0.973	0.173	0.143	0.951	0.157
Riboflavin	7	1	1.441	1.043	0.758	0.801	0.934	0.770
Betaine	7	1	1.098	0.807	0.427	0.043	0.277	0.054
Thiamine	7	1	1.096	0.891	0.976	0.693	0.459	0.660
Methionine	7	1	1.559	0.354	0.640	0.011	0.006	0.006
Pyridoxal 5'-phosphate	7	1	2.659	0.934	0.045	0.032	0.713	0.021
Folic acid	7	1	1.131	0.622	0.424	0.090	0.426	0.105
Dihydrofolic acid	7	1	1.710	1.168	0.727	0.811	0.929	0.796
Serine	7	1	1.115	0.640	0.368	0.015	0.437	0.067
5-methyltetrahydrofolate	7	1	1.572	1.090	0.202	0.304	0.787	0.332
S-adenosylhomocysteine	7	1	1.472	1.065	0.123	0.211	0.838	0.167
Methylcobalamin	7	1	1.310	1.447	0.738	0.405	0.478	0.320
Homocysteine	7	1	1.866	0.926	0.009	0.004	0.611	0.002
S-adenosylmethionine	7	1	0.664	1.049	0.001	0.006	0.832	0.001
Cystathionine	7	1	1.490	1.044	0.045	0.056	0.906	0.078
Cystine	7	1	1.886	0.450	0.899	0.298	0.062	0.234
SAM/SAH	7	1.189	0.490	1.579	0.006	0.008	0.997	0.007

SAM/SAH ratio was calculated from the s-adenosylmethionine and s-adenosylhomocysteine data.

* p-value is calculated using two-tailed unpaired student t-test.

Targeted metabolomics data is in Supplementary Data 3.

Supplementary Table 3. Cox regression analysis of OCM/tryptophan metabolism genes and *fmo-2* modified strains paraquat stress resistance data

	Experimental		FMO-2 OE		Interact with FMO-2 OE	
	Haz. Ratio	p-value	Haz. Ratio	p-value	Haz. Ratio	p-value
sams-1	1.630	<0.001	0.273	<0.001	2.982	<0.001
mel-32	0.299	<0.001	0.263	<0.001	1.593	0.002
mtrr-1	0.732	0.001	0.323	<0.001	0.874	0.310
Y106G6E.4	0.493	<0.001	0.309	<0.001	1.184	0.200
kmo-1	0.321	<0.001	0.243	<0.001	2.741	<0.001
tdo-2	1.721	<0.001	0.290	<0.001	1.477	<0.001
nkat-1	0.667	<0.001	0.302	<0.001	0.848	0.212

Experimental = effect of RNAi condition on worm stress resistance

FMO-2 OE = effect of overexpressing *fmo-2* on worm stress resistance

Interact with FMO-2 OE = interaction between RNAi and overexpressing *fmo-2* on worm stress resistance

Hazard ratio > 1 = decrease in stress resistance

Hazard ratio < 1 = increase in stress resistance

Supplementary Table 4: Stress resistance analyses against 5 mM paraquat

Strain	Experiment	Food	n	Median	Mean	p-value	% change median	% change mean
KAE9	2	afmd-1 (RNAi)	48	10	13.1	0.058	0%	26%
KAE9	6	afmd-1 (RNAi)	49	13	15.0	0.339	-13%	-7%
KAE9	7	afmd-1 (RNAi)	56	9	12.5	0.631	-18%	-7%
KAE9	8	afmd-1 (RNAi)	92	14	16.3	0.000	40%	30%
KAE9	9	afmd-1 (RNAi)	64	19	17.0	0.011	58%	21%
Wild-type/Control	2	afmd-1 (RNAi)	88	7	6.8	0.001	17%	15%
Wild-type/Control	6	afmd-1 (RNAi)	42	9	10.1	0.802	0%	3%
Wild-type/Control	7	afmd-1 (RNAi)	53	8	7.8	0.227	0%	-5%
Wild-type/Control	8	afmd-1 (RNAi)	74	9	10.4	0.000	29%	38%
Wild-type/Control	9	afmd-1 (RNAi)	70	8	9.5	0.636	0%	4%
KAE9	4	alh-3 (RNAi)	65	6	6.9	0.000	-40%	-29%
KAE9	5	alh-3 (RNAi)	49	4	5.3	0.069	-33%	-13%
KAE9	10	alh-3 (RNAi)	103	18	18.7	0.498	-14%	-2%
Wild-type/Control	4	alh-3 (RNAi)	16	5	5.1	0.000	-17%	-23%
Wild-type/Control	5	alh-3 (RNAi)	31	5	5.5	0.000	0%	16%
Wild-type/Control	10	alh-3 (RNAi)	81	11	12.1	0.206	0%	8%
KAE9	1	atic-1 (RNAi)	50	6	6.2	0.000	-25%	-34%
KAE9	2	atic-1 (RNAi)	67	10	12.4	0.028	0%	19%
KAE9	7	atic-1 (RNAi)	66	6	8.4	0.000	-45%	-37%
KAE9	8	atic-1 (RNAi)	64	12	13.9	0.205	20%	11%
KAE9	9	atic-1 (RNAi)	67	19	17.3	0.004	58%	24%
Wild-type/Control	1	atic-1 (RNAi)	53	5	4.7	0.003	0%	-7%
Wild-type/Control	2	atic-1 (RNAi)	64	6	6.3	0.210	0%	6%
Wild-type/Control	7	atic-1 (RNAi)	33	6	6.7	0.000	-25%	-18%
Wild-type/Control	8	atic-1 (RNAi)	57	7	7.6	0.604	0%	1%
Wild-type/Control	9	atic-1 (RNAi)	57	9	10.0	0.358	13%	10%
KAE9	1	cbs-1 (RNAi)	37	7	7.1	0.001	-13%	-24%
KAE9	4	cbs-1 (RNAi)	43	8	7.7	0.000	-20%	-21%
KAE9	5	cbs-1 (RNAi)	34	6	6.2	0.417	0%	2%
Wild-type/Control	1	cbs-1 (RNAi)	52	6	6.3	0.000	20%	25%

Wild-type/Control	4	cbs-1 (RNAi)	41	7	6.8	0.110	17%	4%
Wild-type/Control	5	cbs-1 (RNAi)	39	6	5.5	0.000	20%	17%
KAE9	1	cth-2 (RNAi)	62	6	6.4	0.000	-25%	-33%
KAE9	4	cth-2 (RNAi)	42	8	7.8	0.000	-20%	-20%
KAE9	5	cth-2 (RNAi)	41	7	7.6	0.000	17%	25%
KAE9	10	cth-2 (RNAi)	76	16	17.5	0.064	-24%	-8%
Wild-type/Control	1	cth-2 (RNAi)	36	6	6.1	0.000	20%	21%
Wild-type/Control	4	cth-2 (RNAi)	73	5	5.2	0.000	-17%	-21%
Wild-type/Control	5	cth-2 (RNAi)	46	5	5.3	0.000	0%	12%
Wild-type/Control	10	cth-2 (RNAi)	81	16	16.9	0.000	45%	50%
KAE9	1	EV (RNAi)	22	8	9.4	N/A	0%	0%
KAE9	2	EV (RNAi)	42	10	10.4	N/A	0%	0%
KAE9	3	EV (RNAi)	48	13	14.5	N/A	0%	0%
KAE9	4	EV (RNAi)	49	10	9.8	N/A	0%	0%
KAE9	5	EV (RNAi)	34	6	6.1	N/A	0%	0%
KAE9	6	EV (RNAi)	39	15	16.1	N/A	0%	0%
KAE9	7	EV (RNAi)	34	11	13.4	N/A	0%	0%
KAE9	8	EV (RNAi)	65	10	12.5	N/A	0%	0%
KAE9	9	EV (RNAi)	75	12	14.0	N/A	0%	0%
KAE9	10	EV (RNAi)	91	21	19.1	N/A	0%	0%
Wild-type/Control	1	EV (RNAi)	61	5	5.0	N/A	0%	0%
Wild-type/Control	2	EV (RNAi)	73	6	5.9	N/A	0%	0%
Wild-type/Control	3	EV (RNAi)	52	8	8.1	N/A	0%	0%
Wild-type/Control	4	EV (RNAi)	66	6	6.6	N/A	0%	0%
Wild-type/Control	5	EV (RNAi)	24	5	4.7	N/A	0%	0%
Wild-type/Control	6	EV (RNAi)	55	9	9.8	N/A	0%	0%
Wild-type/Control	7	EV (RNAi)	56	8	8.2	N/A	0%	0%
Wild-type/Control	8	EV (RNAi)	78	7	7.5	N/A	0%	0%
Wild-type/Control	9	EV (RNAi)	44	8	9.1	N/A	0%	0%
Wild-type/Control	10	EV (RNAi)	72	11	11.2	N/A	0%	0%
KAE9	2	kmo-1 (RNAi)	36	12	11.7	0.038	20%	12%
KAE9	3	kmo-1 (RNAi)	57	20	18.6	0.006	54%	28%
KAE9	7	kmo-1 (RNAi)	45	15	13.6	0.956	36%	2%
KAE9	10	kmo-1 (RNAi)	95	21	20.6	0.922	0%	8%

Wild-type/Control	2	kmo-1 (RNAi)	61	7	7.1	0.000	17%	19%
Wild-type/Control	3	kmo-1 (RNAi)	63	16	15.2	0.000	100%	86%
Wild-type/Control	7	kmo-1 (RNAi)	58	9	9.7	0.001	13%	19%
Wild-type/Control	10	kmo-1 (RNAi)	99	21	20.5	0.000	91%	83%
KAE9	1	mel-32 (RNAi)	37	9	9.1	0.243	13%	-3%
KAE9	3	mel-32 (RNAi)	66	22	20.1	0.000	69%	39%
KAE9	4	mel-32 (RNAi)	36	7	8.1	0.000	-30%	-17%
KAE9	8	mel-32 (RNAi)	36	23	19.8	0.000	130%	59%
Wild-type/Control	1	mel-32 (RNAi)	51	6	5.6	0.000	20%	12%
Wild-type/Control	3	mel-32 (RNAi)	66	16	16.0	0.000	100%	97%
Wild-type/Control	4	mel-32 (RNAi)	36	7	7.2	0.000	17%	9%
Wild-type/Control	8	mel-32 (RNAi)	88	15	14.9	0.000	114%	99%
KAE9	2	mtrr-1 (RNAi)	61	12	14.9	0.001	20%	43%
KAE9	7	mtrr-1 (RNAi)	44	9	11.0	0.054	-18%	-18%
KAE9	8	mtrr-1 (RNAi)	72	15	16.0	0.003	50%	28%
KAE9	9	mtrr-1 (RNAi)	70	21	19.0	0.000	75%	36%
Wild-type/Control	2	mtrr-1 (RNAi)	77	7	6.6	0.007	17%	11%
Wild-type/Control	7	mtrr-1 (RNAi)	39	7	7.6	0.104	-13%	-8%
Wild-type/Control	8	mtrr-1 (RNAi)	77	9	9.9	0.000	29%	32%
Wild-type/Control	9	mtrr-1 (RNAi)	42	8	9.8	0.312	0%	7%
KAE9	2	nkat-1 (RNAi)	39	12	12.2	0.017	20%	16%
KAE9	7	nkat-1 (RNAi)	71	13	14.9	0.143	18%	12%
KAE9	8	nkat-1 (RNAi)	63	14	15.2	0.019	40%	22%
KAE9	9	nkat-1 (RNAi)	73	23	21.3	0.000	92%	52%
Wild-type/Control	2	nkat-1 (RNAi)	79	6	6.8	0.001	0%	15%
Wild-type/Control	7	nkat-1 (RNAi)	49	7	7.5	0.034	-13%	-8%
Wild-type/Control	8	nkat-1 (RNAi)	63	10	9.6	0.000	43%	28%
Wild-type/Control	9	nkat-1 (RNAi)	79	9	10.6	0.044	13%	16%
KAE9	3	sams-1 (RNAi)	66	8	7.9	0.000	-38%	-45%
KAE9	6	sams-1 (RNAi)	27	8	8.3	0.000	-47%	-48%
KAE9	10	sams-1 (RNAi)	106	11	9.8	0.000	-48%	-49%
Wild-type/Control	3	sams-1 (RNAi)	72	7	7.4	0.000	-13%	-10%

Wild-type/Control	6	sams-1 (RNAi)	45	7	7.6	0.000	-22%	-23%
Wild-type/Control	10	sams-1 (RNAi)	111	11	9.5	0.000	0%	-16%
KAE9	3	tdo-2 (RNAi)	44	8	9.0	0.000	-38%	-38%
KAE9	8	tdo-2 (RNAi)	49	8	9.5	0.002	-20%	-24%
KAE9	10	tdo-2 (RNAi)	79	11	11.6	0.000	-48%	-39%
Wild-type/Control	3	tdo-2 (RNAi)	64	7	7.1	0.000	-13%	-13%
Wild-type/Control	8	tdo-2 (RNAi)	81	6	5.9	0.000	-14%	-21%
Wild-type/Control	10	tdo-2 (RNAi)	65	11	9.2	0.000	0%	-18%
KAE9	2	Y106G6E.4 (RNAi)	65	12	13.6	0.003	20%	30%
KAE9	3	Y106G6E.4 (RNAi)	54	16	17.1	0.059	23%	18%
KAE9	7	Y106G6E.4 (RNAi)	67	13	13.9	0.730	18%	4%
KAE9	9	Y106G6E.4 (RNAi)	96	21	21.3	0.000	75%	52%
Wild-type/Control	2	Y106G6E.4 (RNAi)	77	7	6.8	0.002	17%	14%
Wild-type/Control	3	Y106G6E.4 (RNAi)	62	9	10.5	0.000	13%	29%
Wild-type/Control	7	Y106G6E.4 (RNAi)	52	7	7.4	0.005	-13%	-10%
Wild-type/Control	9	Y106G6E.4 (RNAi)	80	19	16.2	0.000	138%	77%

KAE9 = FMO-2 OE

Cox regression analysis is in Supplementary Data 4.

Supplementary Table 5: Lifespan analyses

Strain	Experiment	Food	n	Median	Mean	p-value	% change median	% change mean
KAE9	6	afmd-1 (RNAi)	130	29	29.4	0.017	-12%	-9%
KAE9	7	afmd-1 (RNAi)	114	35	33.9	0.019	6%	2%
KAE9	13	afmd-1 (RNAi)	85	28	26.7	0.016	0%	-6%
VC1668	6	afmd-1 (RNAi)	93	27	27.2	0.861	0%	0%
VC1668	7	afmd-1 (RNAi)	105	31	28.8	0.441	11%	1%
VC1668	13	afmd-1 (RNAi)	103	22	23.6	0.016	-12%	-5%
Wild-type/Control	6	afmd-1 (RNAi)	132	29	28.0	0.241	0%	-1%
Wild-type/Control	7	afmd-1 (RNAi)	127	31	30.6	0.001	0%	4%
Wild-type/Control	13	afmd-1 (RNAi)	100	28	25.7	0.091	0%	-4%
KAE9	1	alh-3 (RNAi)	131	31	28.9	0.000	0%	-5%
KAE9	4	alh-3 (RNAi)	118	37	38.0	0.168	0%	-1%
KAE9	8	alh-3 (RNAi)	129	35	34.2	0.000	-10%	-8%
VC1668	1	alh-3 (RNAi)	131	28	27.3	0.306	0%	1%
VC1668	4	alh-3 (RNAi)	107	33	32.5	0.514	0%	-1%
VC1668	8	alh-3 (RNAi)	129	31	30.9	0.524	0%	1%
Wild-type/Control	1	alh-3 (RNAi)	134	26	26.4	0.000	-7%	-5%
Wild-type/Control	4	alh-3 (RNAi)	124	33	34.1	0.051	0%	7%
Wild-type/Control	8	alh-3 (RNAi)	137	33	31.9	0.915	0%	1%
KAE9	1	atic-1 (RNAi)	124	31	28.7	0.029	0%	-6%
KAE9	4	atic-1 (RNAi)	117	33	30.6	0.000	-11%	-20%
KAE9	8	atic-1 (RNAi)	141	33	32.6	0.012	-15%	-13%
VC1668	1	atic-1 (RNAi)	129	31	28.5	0.003	11%	6%
VC1668	4	atic-1 (RNAi)	112	33	33.4	0.262	0%	2%
VC1668	8	atic-1 (RNAi)	109	33	31.4	0.020	6%	3%

Wild-type/Control	1	atic-1 (RNAi)	137	28	26.4	0.135	0%	-5%
Wild-type/Control	4	atic-1 (RNAi)	108	33	32.5	0.107	0%	2%
Wild-type/Control	8	atic-1 (RNAi)	137	33	31.7	0.168	0%	0%
KAE9	1	cbs-1 (RNAi)	66	33	32.6	0.046	6%	7%
KAE9	3	cbs-1 (RNAi)	101	37	35.5	0.874	0%	-2%
KAE9	8	cbs-1 (RNAi)	135	35	37.1	0.689	-10%	0%
VC1668	1	cbs-1 (RNAi)	129	26	25.9	0.057	-7%	-4%
VC1668	3	cbs-1 (RNAi)	118	33	33.2	0.001	10%	6%
VC1668	8	cbs-1 (RNAi)	140	33	31.9	0.011	6%	4%
Wild-type/Control	1	cbs-1 (RNAi)	128	31	29.6	0.004	11%	6%
Wild-type/Control	3	cbs-1 (RNAi)	115	30	31.5	0.861	-9%	-1%
Wild-type/Control	8	cbs-1 (RNAi)	138	33	34.9	0.000	0%	10%
KAE9	1	cth-2 (RNAi)	131	31	29.8	0.211	0%	-2%
KAE9	3	cth-2 (RNAi)	109	33	34.3	0.046	-11%	-6%
KAE9	8	cth-2 (RNAi)	144	33	32.3	0.000	-15%	-13%
KAE9	9	cth-2 (RNAi)	142	29	27.8	0.000	-12%	-15%
VC1668	1	cth-2 (RNAi)	127	24	22.8	0.000	-14%	-16%
VC1668	3	cth-2 (RNAi)	114	30	27.6	0.000	0%	-12%
VC1668	8	cth-2 (RNAi)	109	26	26.8	0.000	-16%	-12%
VC1668	9	cth-2 (RNAi)	135	25	23.8	0.000	-7%	-12%
Wild-type/Control	1	cth-2 (RNAi)	145	26	25.1	0.000	-7%	-10%
Wild-type/Control	3	cth-2 (RNAi)	110	33	31.4	0.743	0%	-2%
Wild-type/Control	8	cth-2 (RNAi)	157	26	27.3	0.000	-21%	-14%
Wild-type/Control	9	cth-2 (RNAi)	172	25	23.8	0.000	-7%	-12%
KAE9	1	EV (RNAi)	128	31	30.4	N/A	0%	0%
KAE9	2	EV (RNAi)	130	34	32.9	N/A	0%	0%
KAE9	3	EV (RNAi)	118	37	36.3	N/A	0%	0%
KAE9	4	EV (RNAi)	119	37	38.3	N/A	0%	0%
KAE9	5	EV (RNAi)	110	31	29.9	N/A	0%	0%

KAE9	6	EV (RNAi)	124	33	32.2	N/A	0%	0%
KAE9	7	EV (RNAi)	118	33	33.3	N/A	0%	0%
KAE9	8	EV (RNAi)	134	39	37.2	N/A	0%	0%
KAE9	9	EV (RNAi)	145	33	32.7	N/A	0%	0%
KAE9	10	EV (RNAi)	56	29	29.4	N/A	0%	0%
KAE9	11	EV (RNAi)	76	30	28.6	N/A	0%	0%
KAE9	12	EV (RNAi)	118	31	30.2	N/A	0%	0%
KAE9	13	EV (RNAi)	99	28	28.4	N/A	0%	0%
VC1668	1	EV (RNAi)	128	28	27.0	N/A	0%	0%
VC1668	2	EV (RNAi)	140	27	27.0	N/A	0%	0%
VC1668	3	EV (RNAi)	110	30	31.3	N/A	0%	0%
VC1668	4	EV (RNAi)	114	33	32.9	N/A	0%	0%
VC1668	5	EV (RNAi)	123	28	26.6	N/A	0%	0%
VC1668	6	EV (RNAi)	91	27	27.1	N/A	0%	0%
VC1668	7	EV (RNAi)	99	28	28.3	N/A	0%	0%
VC1668	8	EV (RNAi)	116	31	30.6	N/A	0%	0%
VC1668	9	EV (RNAi)	134	27	27.1	N/A	0%	0%
VC1668	10	EV (RNAi)	68	29	28.1	N/A	0%	0%
VC1668	11	EV (RNAi)	79	28	26.7	N/A	0%	0%
VC1668	12	EV (RNAi)	112	27	26.4	N/A	0%	0%
VC1668	13	EV (RNAi)	106	25	24.9	N/A	0%	0%
Wild-type/Control	1	EV (RNAi)	127	28	27.8	N/A	0%	0%
Wild-type/Control	2	EV (RNAi)	123	29	27.4	N/A	0%	0%
Wild-type/Control	3	EV (RNAi)	104	33	31.9	N/A	0%	0%
Wild-type/Control	4	EV (RNAi)	122	33	32.0	N/A	0%	0%
Wild-type/Control	5	EV (RNAi)	118	31	28.3	N/A	0%	0%
Wild-type/Control	6	EV (RNAi)	139	29	28.4	N/A	0%	0%
Wild-type/Control	7	EV (RNAi)	141	31	29.4	N/A	0%	0%
Wild-type/Control	8	EV (RNAi)	139	33	31.7	N/A	0%	0%
Wild-type/Control	9	EV (RNAi)	132	27	27.2	N/A	0%	0%
Wild-type/Control	10	EV (RNAi)	63	29	27.5	N/A	0%	0%
Wild-type/Control	11	EV (RNAi)	79	28	25.9	N/A	0%	0%
Wild-type/Control	12	EV (RNAi)	121	27	26.4	N/A	0%	0%
Wild-type/Control	13	EV (RNAi)	101	28	26.7	N/A	0%	0%

KAE9	6	kmo-1 (RNAi)	125	27	27.4	0.000	-18%	-15%
KAE9	7	kmo-1 (RNAi)	122	31	28.4	0.000	-6%	-15%
KAE9	13	kmo-1 (RNAi)	186	25	25.0	0.000	-11%	-12%
VC1668	6	kmo-1 (RNAi)	91	25	24.8	0.000	-7%	-9%
VC1668	7	kmo-1 (RNAi)	92	26	25.6	0.000	-7%	-10%
VC1668	13	kmo-1 (RNAi)	96	20	21.3	0.000	-20%	-15%
Wild-type/Control	6	kmo-1 (RNAi)	133	23	23.1	0.000	-21%	-19%
Wild-type/Control	7	kmo-1 (RNAi)	135	26	25.5	0.000	-16%	-13%
Wild-type/Control	13	kmo-1 (RNAi)	96	20	21.3	0.000	-29%	-20%
KAE9	2	mel-32 (RNAi)	63	36	36.1	0.008	6%	10%
KAE9	5	mel-32 (RNAi)	97	35	33.0	0.000	13%	10%
KAE9	10	mel-32 (RNAi)	60	38	38.8	0.000	31%	32%
VC1668	2	mel-32 (RNAi)	129	27	26.3	0.187	0%	-3%
VC1668	5	mel-32 (RNAi)	114	28	27.8	0.226	0%	5%
VC1668	10	mel-32 (RNAi)	66	29	28.0	0.718	0%	0%
Wild-type/Control	2	mel-32 (RNAi)	130	29	27.6	0.456	0%	1%
Wild-type/Control	5	mel-32 (RNAi)	121	31	28.0	0.653	0%	-1%
Wild-type/Control	10	mel-32 (RNAi)	63	26	25.9	0.009	-10%	-6%
KAE9	2	mtrr-1 (RNAi)	127	32	30.0	0.000	-6%	-9%
KAE9	3	mtrr-1 (RNAi)	114	37	35.6	0.234	0%	-2%
KAE9	5	mtrr-1 (RNAi)	123	31	29.1	0.232	0%	-3%
VC1668	2	mtrr-1 (RNAi)	129	27	26.0	0.000	0%	-4%
VC1668	3	mtrr-1 (RNAi)	117	30	31.3	0.933	0%	0%
VC1668	5	mtrr-1 (RNAi)	112	26	25.4	0.030	-7%	-5%
Wild-type/Control	2	mtrr-1 (RNAi)	132	27	26.7	0.002	-7%	-3%
Wild-type/Control	3	mtrr-1 (RNAi)	109	33	32.4	1.000	0%	2%
Wild-type/Control	5	mtrr-1 (RNAi)	123	28	26.4	0.001	-10%	-7%

KAE9	6	nkat-1 (RNAi)	125	35	34.4	0.016	6%	7%
KAE9	7	nkat-1 (RNAi)	108	35	35.6	0.000	6%	7%
KAE9	11	nkat-1 (RNAi)	75	32	30.7	0.037	7%	8%
KAE9	13	nkat-1 (RNAi)	92	28	27.6	0.162	0%	-3%
VC1668	6	nkat-1 (RNAi)	109	27	27.6	0.894	0%	2%
VC1668	7	nkat-1 (RNAi)	100	28	28.8	0.432	0%	2%
VC1668	11	nkat-1 (RNAi)	77	28	27.6	0.098	0%	3%
VC1668	13	nkat-1 (RNAi)	115	25	25.2	0.828	0%	1%
Wild-type/Control	6	nkat-1 (RNAi)	131	27	27.5	0.027	-7%	-3%
Wild-type/Control	7	nkat-1 (RNAi)	65	31	30.5	0.097	0%	4%
Wild-type/Control	11	nkat-1 (RNAi)	67	28	27.7	0.005	0%	7%
Wild-type/Control	13	nkat-1 (RNAi)	102	28	25.4	0.025	0%	-5%
KAE9	12	sams-1 (RNAi)	55	31	30.8	0.584	0%	2%
KAE9	9	sams-1 (RNAi)	146	35	34.8	0.001	6%	6%
KAE9	11	sams-1 (RNAi)	70	28	28.5	0.517	-13%	0%
VC1668	9	sams-1 (RNAi)	135	31	31.5	0.000	15%	16%
VC1668	11	sams-1 (RNAi)	81	28	27.5	0.052	0%	3%
VC1668	12	sams-1 (RNAi)	113	27	28.2	0.002	0%	7%
Wild-type/Control	9	sams-1 (RNAi)	151	35	35.3	0.000	30%	30%
Wild-type/Control	11	sams-1 (RNAi)	59	30	29.2	0.000	7%	12%
Wild-type/Control	12	sams-1 (RNAi)	119	29	30.0	0.000	7%	14%
KAE9	6	tdo-2 (RNAi)	131	35	36.1	0.000	6%	12%
KAE9	7	tdo-2 (RNAi)	139	38	38.1	0.000	15%	14%
KAE9	13	tdo-2 (RNAi)	96	37	36.6	0.000	32%	29%
VC1668	6	tdo-2 (RNAi)	74	29	29.0	0.003	7%	7%
VC1668	7	tdo-2 (RNAi)	100	35	33.5	0.000	25%	18%
VC1668	13	tdo-2 (RNAi)	102	28	26.1	0.001	12%	5%

Wild-type/Control	6	tdo-2 (RNAi)	129	35	35.3	0.000	21%	24%
Wild-type/Control	7	tdo-2 (RNAi)	137	38	37.3	0.000	23%	27%
Wild-type/Control	13	tdo-2 (RNAi)	101	33	31.1	0.000	18%	16%
KAE9	1	Y106G6E.4 (RNAi)	131	31	29.4	0.190	0%	9%
KAE9	4	Y106G6E.4 (RNAi)	126	37	38.2	0.504	0%	0%
KAE9	8	Y106G6E.4 (RNAi)	122	39	36.5	0.584	0%	-2%
VC1668	1	Y106G6E.4 (RNAi)	69	28	28.2	0.291	0%	4%
VC1668	4	Y106G6E.4 (RNAi)	123	33	33.0	0.926	0%	0%
VC1668	8	Y106G6E.4 (RNAi)	122	33	31.4	0.024	6%	3%
Wild-type/Control	1	Y106G6E.4 (RNAi)	149	31	27.6	0.644	11%	-1%
Wild-type/Control	4	Y106G6E.4 (RNAi)	119	33	33.8	0.030	0%	6%
Wild-type/Control	8	Y106G6E.4 (RNAi)	119	33	31.4	0.477	0%	-1%

KAE9 = FMO-2 OE
VC1668 = FMO-2 KO

Cox regression analysis in Supplementary Data 5.

Supplementary Table 6: Gene expression data used in the computational model

	Reactions	Genes used in the model projection	RT-PCR fold change in FMO-2 OE (normalized to the wild-type)	RT-PCR fold change in FMO-2 KO (normalized to the wild-type)
Reaction 1 (R1):	met --> sam	*sams-1*	1.20	6.80
Reaction 2 (R2):	sam --> sah	*mtrr-1*	1.22	1.61
Reaction 3 (R3):	sah --> hcy	*ahcy-1*	2.05	4.48
Reaction 4 (R4):	hcy + 5mthf --> met + thf	*metr-1*	1.62	3.46
Reaction 5 (R5):	hcy --> cyst	*cbs-1*	1.22	1.14
Reaction 6 (R6):	cyst --> cys	*cth-2*	1.95	3.36
Reaction 7 (R7):	thf --> 5,10thf	*mel-32*	1.13	2.37
Reaction 8 (R8):	5,10thf --> 5mthf	*mthf-1*	1.31	2.64
Reaction 9 (R9):	met input (methionine transport)	n/a (fold change assumed to be 1)	1.00	1.00
Reaction 10 (R10):	thf input (folate transport)	n/a (fold change assumed to be 1)	1.00	1.00
Reaction 11 (R11):	sam output	*set-2*	0.99	0.89
Reaction 12 (R12):	cys output	*gcs-1*	1.25	1.32
Reaction 13 (R13):	5,10thf output	*tyms-1*	0.68	0.45

met = methionine
sam = s-adenosylmethionine
sah = s-adenosylhomocysteine
hcy = homocysteine
cyst = cystathionine
cys = cysteine
thf = tetrahydrofolate
5,10thf = 5,10-methylenetetrahydrofolate
5mthf = 5-methyltetrahydrofolate

Supplementary Table 7: Stoichiometric matrix for computational model

	R1	R2	R3	R4	R5	R6	R7	R8	R9	R10	R11	R12	R13
met	-1	0	0	1	0	0	0	0	1	0	0	0	0
sam	1	-1	0	0	0	0	0	0	0	0	-1	0	0
sah	0	1	-1	0	0	0	0	0	0	0	0	0	0
hcy	0	0	1	-1	-1	0	0	0	0	0	0	0	0
cyst	0	0	0	0	1	-1	0	0	0	0	0	0	0
cys	0	0	0	0	0	1	0	0	0	0	0	-1	0
thf	0	0	0	1	0	0	-1	0	0	1	0	0	0
5,10thf	0	0	0	0	0	0	1	-1	0	0	0	0	-1
5mthf	0	0	0	-1	0	0	0	1	0	0	0	0	0

met = methionine
sam = s-adenosylmethionine
sah = s-adenosylhomocysteine
hcy = homocysteine
cyst = cystathionine
cys = cysteine
thf = tetrahydrofolate
5,10thf = 5,10-methylenetetrahydrofolate
5mthf = 5-methyltetrahydrofolate

R = reaction

Supplementary Table 8: S-adenosylmethionine (SAM) supplementation lifespan analyses

Strain	Experiment	Food	n	Median	Mean	p-value	% change median	% change mean
KAE9	1	Control	76	30	30.8	N/A	0%	0%
KAE9	2	Control	100	27	27.2	N/A	0%	0%
KAE9	1	2 mM SAM	74	27	27.9	0.001	-10%	-10%
KAE9	2	2 mM SAM	100	27	25.3	0.000	0%	-7%

Supplementary Table 9: FMO-2 enzyme kinetics analyses

Substrate	Km (mM)	kcat (sec-1)	Catalytic Efficiency (sec-1 M-1)
NADPH	2.50 ± 1.24	264 ± 98	105000 ± 65000
Methimazole	1.92 ± 1.14	13.0 ± 6.0	6800 ± 5100
Tryptophan	0.88 ± 0.43	9.7 ± 1.5	11000 ± 5000
Aspartic Acid	N.D.	N.D.	N.D. (Activity starts at 1 mM)
Glutamine	N.D.	N.D.	N.D. (Activity starts at 3 mM)
TMA	N.D.	N.D.	N.D. (Activity starts at 10 mM)
Cysteine	N.D.	N.D.	N.D. (Activity starts at 10 mM)
Phenylalanine	N.D.	N.D.	N.D. (Activity starts at 10 mM)
L-Methionine	N.D.	N.D.	N.D. (Activity starts at 20 mM)
Valine	N.D.	N.D.	N.D. (Activity starts at 30 mM)
β-alanine	N.D.	N.D.	N.D.
uracil	N.D.	N.D.	N.D.
2-heptanone	N.D.	N.D.	N.D.

N.D. = not determined

Supplementary Table 10: Formate supplementation lifespan analyses

Strain	Experiment	Food	n	Median	Mean	p-value	% change median	% change mean
KAE9	1	1 mM formate	64	31	31.9	0.102	-6%	-4%
KAE9	2	1 mM formate	100	39	37.1	0.872	0%	-3%
VC1668	1	1 mM formate	105	29	28.3	0.000	12%	7%
VC1668	2	1 mM formate	100	30	30.7	0.050	0%	7%
Wild-type/Control	1	1 mM formate	54	31	30.6	0.001	7%	8%
Wild-type/Control	2	1 mM formate	99	33	32.6	0.000	0%	10%
KAE9	1	Control	99	33	33.4	N/A	0%	0%
KAE9	2	Control	102	39	38.2	N/A	0%	0%
VC1668	1	Control	105	26	26.6	N/A	0%	0%
VC1668	2	Control	106	30	28.7	N/A	0%	0%
Wild-type/Control	1	Control	97	29	28.2	N/A	0%	0%
Wild-type/Control	2	Control	101	33	29.8	N/A	0%	0%

KAE9 = FMO-2 OE
VC1668 = FMO-2 KO

Supplementary Table 11: Non-FUDR lifespan analysis

Strain	Experiment	Food	n	Median	Mean	p-value	% change median	% change mean
KAE9	1	OP50	73	23	24.7	0.000	15%	29%
KAE9	2	OP50	81	23	25.3	0.000	15%	19%
Wild-type/Control	1	OP50	85	20	19.2	N/A	0%	0%
Wild-type/Control	2	OP50	100	20	21.2	N/A	0%	0%

Supplementary Table 12: qRT-PCR primers.

Genes	Forward Primers (5'-3')	Reverse Primers (5'-3')
sams-1	CAAATTTCTGACGCCGTTCTC	TCCGCAGAGCATAACCATAC
mtrr-1	GGAACTGGAGTCTCGGTATTTC	ACGACATCCAAAGAAGAGAACA
ahcy-1	TGAGACTCTTACTGCTCTCG	GGAGAAGATGTTGCAGGAAG
metr-1	GTCGAAGTGGTCTGATTCTT	GCCATCTCTTCAGGAGTTTC
cbs-1	GCCATCTCATACGCAACA	CAGGGTATGCAAGACAGTATC
cth-2	AGAACCAGAGCAATGGGATATG	GGGTTTCCAGCACGAGAATAA
mel-32	GTACACCAACAACGAGAACA	CAGCCTTGCTTGTGAAGT
mthf-1	CGGTTGTCGAGGAGAAGATAAC	GACAAATCGAGGTGGGAAGAA
set-2	GAAGAAGCAGAAACCGAGAA	CCTCTTCAGATCGTGGTTTG
gcs-1	GGAGGAAAGAGGATTGGAAC	GTAGCCAACAGAACATGGAT
tyms-1	GAGAAGGAACACGAAGAGATG	CGTGGTAAGCAACGGAAT
Primers for RNAi validation		
Y106G6E.4	AACGTCAAACAGAGGCGGTT	TGAGCCCTTCGTGAACTGTG
sams-1	AGCCACATCTGCTATCGCTC	TGTCACGTAGCCATCGAGTT
alh-3	CGGTGGATTCAAGCAATCTGG	TTCACAGAAAAGGCATCACTCAAT
mel-32	TCGCCAAACGTGTTGAGGAG	ACATGAAAGTGATAAAATGGCGGG
cth-2	TCAAGCTTTGAAGATTGCCATTCC	GAGGTTGTGGTGGAGACGAG
mtrr-1	CCGGACTTGGAAGCGAAGAA	ACTATCCCCACACATCCTCGAT
cbs-1	AGCGTCATTTCCTAGACGCC	AGAAGCCAAATAATACGGACCAAGT
atic-1	ATGTTGCTCATCCAGGAGGC	GGTGGAAGAGACGAAGTCCAG
Y45F10D.4 (Reference gene)	GTCGCTTCAAATCAGTTCAGC	GTTCTTGTCAAGTGATCCGACA

Supplementary Table 13: List of worm strains used in this study

Description	Strain	Source	Identifier
Wild type	N2	CGC	N2
fmo-2p::mCherry	[(pCF150) (fmo-2p::mCherry + H2B::GFP) + Cbr-unc-119(+)]	Our lab	Lei01
FMO-2 OE	((eft-3p::fmo-2 + h2b::gfp + Cbr-unc-119(+))	Our lab	KAE9
fmo-2 KO	fmo-2(ok2147)	CGC	VC1668
