# Supplementary material for: FMO rewires metabolism to promote longevity through tryptophan and one carbon metabolism in C. elegans
Source: Nat Commun. 2023 Feb 2;14:562. doi: 10.1038/s41467-023-36181-0 (PMC9894935; doi:10.1038/s41467-023-36181-0)
Supplement: Supplementary file 2 — Description of Additional Supplementary Files [file 41467_2023_36181_MOESM2_ESM.pdf]

Description of Additional Supplementary Files

Supplementary Data 1: Untargeted metabolomics data of the wild-type, FMO-2 OE, and FMO-2 KO strains.

Supplementary Data 2: Statistical analysis of untargeted metabolomics data comparing the wild-type and FMO-2 OE that was used in the pathway enrichment analysis (Linked to Supplemental Table 1).

Supplementary Data 3: Targeted metabolomics data of the wild-type, FMO-2 OE, and FMO-2 KO. The resulting statistical analysis is in Supplemental Table 2.

Supplementary Data 4: Cox regression analysis of stress resistance experiments on the wild-type and FMO-2 OE using 5 Mm paraquat (Linked to Supplemental Table 3).

Supplementary Data 5: Cox regression analysis of lifespan experiments using the wild-type, FMO-2 OE, and FMO-2 KO (Linked with Supplemental Tables 4 and 8).

Supplementary Data 6: Computational model code for flux analysis.

Supplementary Data 7: Untargeted metabolomics of purified FMO2 assay samples. Feature detection by XCMS.
