# Supplementary material for: FMO rewires metabolism to promote longevity through tryptophan and one carbon metabolism in C. elegans
Source: Nat Commun. 2023 Feb 2;14:562. doi: 10.1038/s41467-023-36181-0 (PMC9894935; doi:10.1038/s41467-023-36181-0)
Supplement: Supplementary file 8 — Supplementary Data 6 [file 41467_2023_36181_MOESM8_ESM.docx]

% Supplementary Data 6: Computational model code

S = xlsread('Supplemental_Table_7','B2:N10'); %Reads one carbon metabolism stoichiometric matrix

M = null(S,'r'); %Nullspace of S

fc_ox = xlsread('Supplemental_Table_6.xlsx','D2:D14'); %Reads fmo-2 overexpression relative gene expression fold change

fc_ko = xlsread('Supplemental_Table_6.xlsx','E2:E14'); %Reads fmo-2 knockout relative gene expression fold change

fc_wt = ones(13,1);

for i = (1:length(fc_ox)) %Cut-off for gene expression data

if fc_ox(i) < 1.5 && fc_ox(i) > 0.5

fc_ox(i) = 1;

end

end

for i = (1:length(fc_ko)) %Cut-off for gene expression data

if fc_ko(i) < 1.5 && fc_ko(i) > 0.5

fc_ko(i) = 1;

end

end

a_ox = lsqminnorm(M,fc_ox)'; %relative factor for fmo-2 overexpression strain

a_ko = lsqminnorm(M,fc_ko)'; %relative factor for fmo-2 knockout strain

a_wt = lsqminnorm(M,fc_wt)'; %relative factor for wild type

J_ox = M * a_ox'; %flux prediction for fmo-2 overexpression strain

J_ko = M * a_ko'; %flux prediction for fmo-2 knockout strain

J_wt = M * a_wt'; %flux prediction for wild type

J_rel_ox = J_ox./J_wt; %relative flux prediction for fmo-2 overexpression strain

J_rel_ko = J_ko./J_wt; %relative flux prediction for fmo-2 knockout strain

figure

cdata = [J_rel_ox,J_rel_ko];

xvalues = {'FMO-2 OE','FMO-2 KO'};

yvalues = {'met -> sam','sam -> sah','sah -> hcy','hcy + 5mthf -> met + thf',...

'hcy -> cyst','cyst -> cys','thf -> 5,10thf','5,10thf -> 5mthf','METin',...

'FOLin', 'methylation','cys consumption','pyrimidine synthesis'};

h = heatmap(xvalues,yvalues,cdata);
