# Supplementary material for: FMO rewires metabolism to promote longevity through tryptophan and one carbon metabolism in C. elegans
Source: Nat Commun. 2023 Feb 2;14:562. doi: 10.1038/s41467-023-36181-0 (PMC9894935; doi:10.1038/s41467-023-36181-0)
Supplement: Supplementary file 10 — Reporting Summary [file 41467_2023_36181_MOESM10_ESM.pdf]

nature portfolio

Corresponding author(s): Scott F. Leiser

Last updated by author(s): Jan 17, 2022

Reporting Summary

Nature Portfolio wishes to improve the reproducibility of the work that we publish. This form provides structure for consistency and transparency in reporting. For further information on Nature Portfolio policies, see our Editorial Policies and the Editorial Policy Checklist.

Statistics

For all statistical analyses, confirm that the following items are present in the figure legend, table legend, main text, or Methods section.

n/a	Confirmed	
☐	☒	The exact sample size (*n*) for each experimental group/condition, given as a discrete number and unit of measurement
☐	☒	A statement on whether measurements were taken from distinct samples or whether the same sample was measured repeatedly
☐	☒	The statistical test(s) used AND whether they are one- or two-sided<br>*Only common tests should be described solely by name; describe more complex techniques in the Methods section.*
☐	☒	A description of all covariates tested
☐	☒	A description of any assumptions or corrections, such as tests of normality and adjustment for multiple comparisons
☐	☒	A full description of the statistical parameters including central tendency (e.g. means) or other basic estimates (e.g. regression coefficient) AND variation (e.g. standard deviation) or associated estimates of uncertainty (e.g. confidence intervals)
☐	☒	For null hypothesis testing, the test statistic (e.g. *F*, *t*, *r*) with confidence intervals, effect sizes, degrees of freedom and *P* value noted<br>*Give P values as exact values whenever suitable.*
☒	☐	For Bayesian analysis, information on the choice of priors and Markov chain Monte Carlo settings
☒	☐	For hierarchical and complex designs, identification of the appropriate level for tests and full reporting of outcomes
☒	☐	Estimates of effect sizes (e.g. Cohen's *d*, Pearson's *r*), indicating how they were calculated

Our web collection on statistics for biologists contains articles on many of the points above.

Software and code

Policy information about availability of computer code

Data collection	All images in this study were acquired using Leica Application Suite X software.
Data analysis	Fluorescence was quantified by ImageJ bundled with 64-bit Java 1.8.0. Data were plotted using Microsoft Excel 365, Metaboanalyst 4.0, XCMS 3.16, Agilent MassHunter Quantitative Analysis software, and Graph Pad Prism version 9.1.0. The statistical analyses were done using OASIS 2, Microsoft Excel 365, STATA 14, Metaboanalyst 4.0, and GraphPad Prism version 9.1.0. Computer modeling was done using MATLAB 2018a.

For manuscripts utilizing custom algorithms or software that are central to the research but not yet described in published literature, software must be made available to editors and reviewers. We strongly encourage code deposition in a community repository (e.g. GitHub). See the Nature Portfolio guidelines for submitting code & software for further information.

Data

Policy information about availability of data

All manuscripts must include a data availability statement. This statement should provide the following information, where applicable:

- Accession codes, unique identifiers, or web links for publicly available datasets
- A description of any restrictions on data availability
- For clinical datasets or third party data, please ensure that the statement adheres to our policy

All data are available in the supplementary files, supplemental tables, and source data file. All data will also be available upon request.

Human research participants

Policy information about studies involving human research participants and Sex and Gender in Research.

Reporting on sex and gender	N/A
Population characteristics	N/A
Recruitment	N/A
Ethics oversight	N/A

Note that full information on the approval of the study protocol must also be provided in the manuscript.

Field-specific reporting

Please select the one below that is the best fit for your research. If you are not sure, read the appropriate sections before making your selection.

☒ Life sciences ☐ Behavioural & social sciences ☐ Ecological, evolutionary & environmental sciences

For a reference copy of the document with all sections, see nature.com/documents/nr-reporting-summary-flat.pdf

Life sciences study design

All studies must disclose on these points even when the disclosure is negative.

Sample size	No sample size calculation was performed. These numbers are sufficient based on our previous studies in C. elegans (Leiser SF et al. 2015, Science and Beydoun S et al. 2021, Communications Biology).
Data exclusions	No data were excluded from the analyses.
Replication	Each experiment is completed at least 3 times for primary findings and at least 2 times for all experiments. Replications were successful unless noted.
Randomization	20 - 30 randomly picked gravid adult animals were placed on growth medium plates for a timed egg-lay. Once their progeny reached late L4/ early adult stage, animals were randomly transferred to plates with different treatment conditions. These progeny worms were therefore allocated into experimental groups randomly. The same procedure was used for the metabolomics samples, except the progeny were transferred as eggs.
Blinding	The investigator was blinded to group allocation during data collection and analysis. Another individual labels the experimental plates as numbers, and the investigator gets the number key for experimental groups after data collection and analysis.

Reporting for specific materials, systems and methods

We require information from authors about some types of materials, experimental systems and methods used in many studies. Here, indicate whether each material, system or method listed is relevant to your study. If you are not sure if a list item applies to your research, read the appropriate section before selecting a response.

Materials & experimental systems

n/a	Involved in the study	
☒	☐	Antibodies
☒	☐	Eukaryotic cell lines
☒	☐	Palaeontology and archaeology
☐	☒	Animals and other organisms
☒	☐	Clinical data
☒	☐	Dual use research of concern

Methods

n/a	Involved in the study	
☒	☐	ChIP-seq
☒	☐	Flow cytometry
☒	☐	MRI-based neuroimaging

Animals and other research organisms

Policy information about studies involving animals; ARRIVE guidelines recommended for reporting animal research, and Sex and Gender in Research

Laboratory animals	Caenorhabditis elegans (strains are mentioned in the methods and animals were tested at ages between hatching and end of life). KAE9 (FMO-2 OE), VC1668 (FMO-2 KO), N2 (Wild type)
Wild animals	The study did not involve wild animals.
Reporting on sex	Sex-based analysis was not done because all animals tested were hermaphrodites.
Field-collected samples	The study did not involve samples collected from the field.
Ethics oversight	No ethical approval or guidance was required as only invertebrate species were utilized.

Note that full information on the approval of the study protocol must also be provided in the manuscript.
